# Rapid purity and charge-variant analysis of bispecific antibodies using microfluidic capillary electrophoresis-mass spectrometry

**DOI:** 10.1007/s00216-026-06748-z

**Published:** 2026-08-22

**Authors:** Ruben Cageling, Karin Lubbers-Geuijen, Govert W. Somsen, Kevin Jooß

**Affiliations:** 1 Protein Purification & Characterization Department, Polpharma Biologics Utrecht, Yalelaan 46, 3584 CM Utrecht, The Netherlands; 2 Division of BioAnalytical Chemistry, Department of Chemistry and Pharmaceutical Sciences, Amsterdam Institute for Molecular and Life Sciences (AIMMS), Vrije Universiteit Amsterdam, de Boelelaan 1085, 1081 HV Amsterdam, The Netherlands; 3 Centre for Analytical Sciences Amsterdam (CASA), Amsterdam, The Netherlands

**Keywords:** High throughput, Capillary zone electrophoresis, Homodimers, Clone screening, Downstream purification

## Abstract

Bispecific antibody (BsAb) therapeutics have the ability to bind two targets simultaneously, which opens up new pathways for disease treatment. BsAbs consist of distinct heavy and light chains that need to be assembled correctly to form the appropriate heterodimer. However, during development and production, misassembly of chains may occur, which affects BsAb purity, and therefore needs to be monitored. Here, we demonstrate the usefulness of microfluidic capillary electrophoresis-mass spectrometry (MCE-MS) for rapid purity and proteoform profiling of BsAb samples. MCE was capable of resolving homodimeric antibody species as well as charge variants from a model BsAb, while MS detection provided identification of these BsAb charge variants and glycoforms. A low-resolution MCE-MS method allowed quantitative determination of homodimer content in less than 3 min, whereas a high-resolution MCE-MS method delivered the detailed charge-variant and glycoform profile of the BsAb therapeutic within 15 min. We applied both methods in tandem for clone screening and downstream purification monitoring of large biosimilar BsAb sample sets. First, the homodimer-heterodimer ratio of BsAb cell line samples and purification process intermediates was established using the fast MCE-MS method. Next, based on the determined BsAb purity, samples of interest were selected and analyzed with the high-resolution MCE-MS method yielding a quality profile, permitting rational clone selection and optimization of the purification process. Overall, the developed MCE-MS workflow exhibits great potential to accelerate and enhance decision-making during BsAb development and production.

**Graphical Abstract:**

**Figure:**
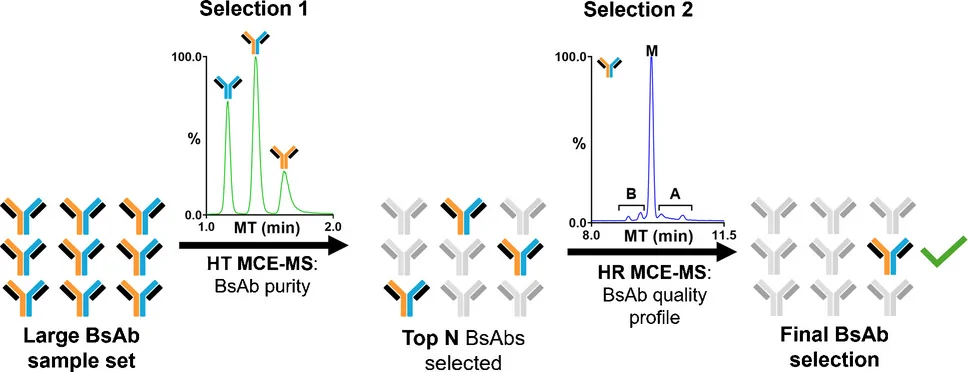

**Supplementary Information:**

The online version contains supplementary material available at https://doi.org/10.1007/s00216-026-06748-z.

## Introduction

Since the first approval of a monoclonal antibody (mAb) as a biotherapeutic in 1986, mAbs have gradually developed into an established class of medicines used across a wide range of clinical indications [1]. Advancements in protein engineering have enabled the development of novel antibody-based therapeutics, including antibody fragments, antibody-drug conjugates (ADCs), and bispecific antibodies (BsAbs). BsAbs differ from conventional mAbs in their ability to simultaneously bind to two different antigens or epitopes. This dual-binding capability can be exploited through various modes of action, such as the concurrent activation of multiple receptors on a single cell or by physically bridging two different cells, e.g., redirecting T-cells towards cancer cells [2]. The first bispecific antibody was approved by the European Medicines Agency (EMA) in 2009; since then, numbers of accepted BsAbs have been steadily growing to more than 20 in 2025 [1]. In addition, by 2024, over 200 BsAbs were in clinical development for oncology alone, highlighting the growing recognition that their dual-targeting functionality addresses unmet needs [3].

Therapeutic BsAbs are often based on immunoglobulin G (IgG) antibodies and typically consist of two different heavy chains (HC_A_ and HC_B_) and two different light chains (LC_A_ and LC_B_) that are all co-expressed within a single cell. Preferably, the chains should assemble into the BsAb (LC_A_-HC_A_-HC_B_-LC_B_), but they might also combine differently, which results in up to nine different undesired Ab variants. For example, if two of the same HCs assemble with two of the same LCs, monospecific antibodies called homodimers are formed (e.g., LC_A_-HC_A_-HC_A_-LC_A_) [4, 5]. These homodimers will only interact with one of the two targets, thereby limiting the effectiveness of the BsAb. To promote the correct assembly of the different chains into the BsAb, several sequence engineering strategies have been applied such as “knobs-into-holes”, re-engineered disulfide bridges (DuetmAb) or by using a common LC [4, 6, 7]. Despite all these innovations, mispaired species such as homodimers are still frequently encountered, which thus requires continuous monitoring of BsAb purity during the various development and production stages. For instance, during cell line development, it is essential to select clones that exhibit both high BsAb productivity and high purity, to prevent extensive, and consequently more costly, downstream purification. During downstream purification, the efficiency of homodimer removal should be monitored, also enabling adjustment of process parameters to achieve high product quality. In the aforementioned stages, often many samples need to be handled, highlighting the need for fast and preferably sensitive analytical methods for BsAb purity assessment.

As mispaired Abs differ in molecular mass from the correctly assembled BsAb, mass spectrometry (MS) has been used to quantify the purity of BsAb samples, sometimes after size exclusion chromatography (SEC) or reversed-phase liquid chromatography (RPLC) [8–13]. As such methods do not or only poorly separate the mispaired Abs from the BsAb prior to MS, quantification might be biased or difficult due to ion suppression effects [14]. Hence, methods that efficiently separate the mispaired Abs from the BsAb prior to MS detection are needed to improve the identification of all Ab variants present in a sample, including those present at low abundance. Accordingly, several studies describe the use of techniques such as mixed mode size exclusion chromatography (mmSEC), ion exchange chromatography (IEX), and capillary zone electrophoresis (CZE) coupled to MS to separate and identify all Ab species present in BsAb samples [5, 14–17].

Although BsAb purity is an essential quality aspect to monitor during development and production, the methods described above do not assess other critical quality attributes (CQAs) of the BsAb, such as glycosylation and charge variants, and therefore miss important aspects of the overall quality of the BsAb. These CQAs can be monitored by employing charge-variant analysis (CVA) techniques, where impurities are resolved from the main BsAb based on their difference in net charge. CVA techniques such as IEX, CZE, and imaging capillary isoelectric focusing (iCIEF) coupled to MS are reported in literature for evaluating charge variants and/or glycoforms in BsAb samples [18–20]. Conventional CEX- and CE-MS-based methods generally provide high-resolution separations but require relatively long analysis times, typically exceeding 25 min per sample, while the biopharmaceutical industry often requires faster methods for screening large sample sets. Microchip-based iCIEF-MS offers a faster alternative, with analysis times of around 15 min per sample (excluding conditioning steps), but there is still room for improvement in terms of throughput [19]. In addition, the simultaneous application of these methods to BsAb purity assessment has not yet been demonstrated. Ideally, a single method or platform that can rapidly separate both mispaired and charge variants from the BsAb would be of great interest for the biopharmaceutical industry, as this would result in a highly informative assay for BsAb samples across the various phases of development and production.

A promising technique for this purpose is microfluidic capillary electrophoresis coupled to MS (MCE-MS), in which CZE is performed in etched glass channels on a microfluidic chip, followed by on-chip nano electrospray ionization (nanoESI). The microfluidic chip with its integrated nanoESI emitter provides robust MS coupling compared to regular CZE-MS and enables rapid and sensitive analysis of mAbs, requiring only low sample volumes and concentrations [21–25]. As a proof-of-concept, Wu et al*.* touched upon the potential of MCE-MS for BsAb analysis, showing that both charge variants and homodimers of a BsAb can be separated and identified by MCE-MS within 15 min [22]. However, the close migration of the homodimer charge variants and the BsAb species hindered accurate purity determination, highlighting the need for improved separation performance.

In this study, we further explore and expand the capabilities of MCE-MS for rapid assessment of the purity and charge-variant content of BsAb samples. The developed methods are evaluated using samples obtained during the development of a BsAb biosimilar, which adopts a conventional IgG format featuring two distinct HCs and two identical LCs. Due to engineered mutations in the HC sequences, the BsAb and one of its corresponding homodimers exhibit a relatively low isoelectric point (pI of 6.5 and 5.7, respectively) as compared to most common mAbs (pI typically ≥ 7.5), which provides an additional analytical challenge [26]. In order to improve the resolution, speed, and sensitivity of the method, attention was paid to employing a chip with a short separation channel and to the background electrolyte (BGE). We established two complementary methods that can be used in series on the same system: a high-throughput method for rapid BsAb purity quantification of large sample sets, followed by a high-resolution method for detailed charge variants and glycoform analysis on a selected subset of the samples based on BsAb purity. The applicability of the dual-method approach was examined in two case studies: (i) a BsAb biosimilar clone screening focusing on the selection of cell lines producing the highest BsAb purity, titer, and quality, and (ii) monitoring of BsAb purity and quality progression during downstream processing (DSP).

## Materials and methods

### Materials

ZipChip High Resolution Native (HRN) chip, High Speed (HS) chip, and ZipChip CVA kit (includes BGE and sample diluent) were obtained from Repligen Corporation (Waltham, MA, USA). Acetic acid (Optima, LC-MS grade) and Zeba spin desalting plates (7K MWCO) were purchased from Fisher Scientific (Landsmeer, The Netherlands). Octet Protein A biosensors and Octet Sample Diluent were obtained from Sartorius (Göttingen, Germany). Phosphate-buffered saline (PBS) was acquired from Lonza (Walkersville, MD, USA). Downstream purification intermediate samples were collected and provided by the DSP department at Polpharma Biologics Utrecht B.V. The originator BsAb was acquired by Polpharma Biologics Utrecht B.V.

### BsAb and homodimer cell line development

A BsAb featuring a common LC and two distinct HCs (HC_A_ and HC_B_) was used as a model protein for this study. For the current study, three separate cell lines were set up. The first cell line was transfected with DNA encoding for the LC, HC_A_, and HC_B_ to produce the BsAb. From this starting cell line, 32 different clonal cell lines were derived and used for a biosimilar clone screening study. The two other cell lines were transfected with DNA corresponding to either common LC and HC_A_ (cell line HD_A-A_) or with common LC and HC_B_ (cell line HD_B-B_) with the purpose of producing homodimer variants, which are potential impurities of the target BsAb. After a 7-day fed-batch cell culture, all cell lines were harvested and centrifuged at 300 × g for 5 min, followed by a centrifugation step at 4000 × *g* for 10 min. The resulting cell culture supernatants of the 32 clonal cell lines were subjected to titer determination (see section “Sample preparation”). The cell culture supernatants of the homodimer cell lines were Protein A purified and filtered, resulting in two samples of the pure homodimers.

### Sample preparation

For MCE-MS method development, calibration curves were generated for each Ab variant by individually diluting the homodimers and the originator BsAb to a stock concentration of 100 µg/mL using ZipChip CVA diluent. Working solutions of 50, 25, and 5 µg/mL were prepared by independent dilution of the stock concentration with the same diluent. Lower concentrations of 1 and 0.1 µg/mL were obtained through serial dilution starting from the 5 µg/mL solution. Separately, spiked samples were prepared by diluting the homodimer and the originator BsAb to a concentration of 1000 µg/mL with ZipChip CVA diluent. Next the samples were mixed in the desired ratio (e.g., 20:60:20 HD_A-A_-BsAb-HD_B-B_) and diluted to a final concentration of 100 µg/mL with ZipChip CVA diluent. The stock concentrations used for both the calibration curves and the spiked samples were verified using an Agilent Biotek Synergy H1 plate reader (Agilent, Middelburg, the Netherlands) operated at 280 nm. Samples used for calibration curves and spiking experiments were measured in triplicate, whereas clone screening and DSP samples were analyzed as single injections.

The 32 biosimilar clone screening cell culture supernatants were subjected to titer determination by bio-layer interferometry (BLI) using an Octet RED96e system (Sartorius, Göttingen, Germany) with Octet Protein A biosensors. For BLI, a calibration curve was constructed between 1.25 and 80 µg/mL using the originator BsAb, which was diluted in a mixture of Octet Sample Diluent and PBS (1:10 v/v) and used to determine the titer of the samples. The samples were diluted 100× in the diluted Octet Sample Diluent prior to BLI analysis. Afterwards, the cell culture supernatants were directly diluted in ZipChip CVA diluent to a final concentration of 100 µg/mL, without prior Protein A purification.

The downstream purification intermediate samples contained high salt concentrations and were therefore buffer exchanged into ZipChip CVA diluent. Buffer exchange was performed using a Zeba Spin Desalting Plate in a plate centrifuge according to vendor protocol. The sample concentrations after buffer exchange were measured using the plate reader and diluted to a final concentration of 100 µg/mL using ZipChip CVA diluent.

### MCE-MS analysis

Microfluidic CE-MS was performed using a ZipChip Ti interface and ZipChip autosampler (Repligen Corporation, Waltham, MA, USA) coupled to an Orbitrap Exploris 240 MS (Thermo Fisher Scientific, Bremen, Germany). For the high-resolution method, an HRN chip was used with ZipChip CVA BGE, pH 5.0 (adjusted from pH 5.5 with acetic acid). The following MCE parameters were applied: field strength, 500 V/cm; injection volume, 1 nL; pressure assist time, 0.0 min; run time, 15 min; BGE refresh and sample injection step, 5 min. The high-throughput method utilizes an HS chip and the ZipChip CVA BGE, pH 4.5 (adjusted with acetic acid). The following MCE parameters were applied: field strength, 1000 V/cm; injection volume, 1.5 nL; pressure assist time, 0.0 min; run time, 3 min; BGE refresh and sample injection step, 1.6 min. The following MS parameters were applied for both methods: pressure mode, intact protein – standard pressure; sheath gas, 2 psi; ion transfer tube temperature, 300 °C; mass range, 2500–8000 *m/z*; RF lens, 200%; normalized AGC target, 100%; maximum injection time, auto; microscans, 3; source fragmentation, 135 V; resolution at 200 *m/z*, 30,000 (high-resolution method) or 15,000 (high-throughput method).

### Data analysis

MCE-MS data were acquired and processed with Chromeleon 7.3.2 (Thermo Fisher Scientific). The electropherograms were visualized as base peak electropherograms (BPEs), which were smoothed using a 7-point moving average filter. For quantification of the BsAb and homodimers, the peaks in the total ion electropherograms (TIEs) were integrated and the resulting peak areas were used to construct calibration curves. The slopes of the calibration curves of the homodimers were compared to the slope of the BsAb to generate response factors (RFs) for each homodimer. The RFs were used to correct the peak area of the homodimers. The corrected peak area was used to determine the fractional abundance of each Ab variant in a sample. For the clone screening samples, an additional correction factor (matrix factor, MF) was applied for each Ab variant to correct for potential matrix effects, since the samples were directly diluted without prior Protein A purification. The MFs were obtained by comparing the fractional abundances of a representative protein A purified and diluted cell culture supernatant BsAb sample. The calculations pertaining to the RFs and MFs are shown in section S1.

The raw data files obtained from the clone screening and downstream processing applications were exported to ProSight Native 1.026086 (Thermo Fisher Scientific) for deconvolution using the following settings: output mass range, 140,000 to 152,000 Da; overlapping sliding windows mode; time to average, 0.11 min; time to slide average, 0.02 min; deconvolution algorithm, kDecon, binning mass tolerance, 15 ppm; minimum spectral observations, 3; peak picker mode, Gaussian with 0.1 FWHM; minimum no. of charge states, 4; minimum no. of consecutive charges, 4; charge state range, 7–50. The resulting masses were exported to Excel and proteoforms were tentatively assigned if the deconvoluted mass was within a 5 Da (~ 34 ppm) of the expected mass based on the primary amino acid sequence of the BsAb together with the expected migration behavior. The fractional abundance of glycoforms was calculated by dividing the MS area of a specific glycoform (obtained after deconvolution) over the sum of the MS areas of all glycoforms. The relative abundance of charge-migrating proteoforms was calculated by dividing the MS area of that proteoform (having G0F + G0F as glycans) over the MS area of the main G0F + G0F glycoform. Only proteoforms with a relative abundance of ≥ 0.5% were considered. The clone selection was performed as described in [21]. For the scoring, the BsAb purity scores were multiplied with a weighing factor of 2, since high purity was considered to be the most important CQA for this screening. All calculations and figures were made using Microsoft Excel and Graphpad Prism 10.0.

## Results and discussion

**Fig. 1:**
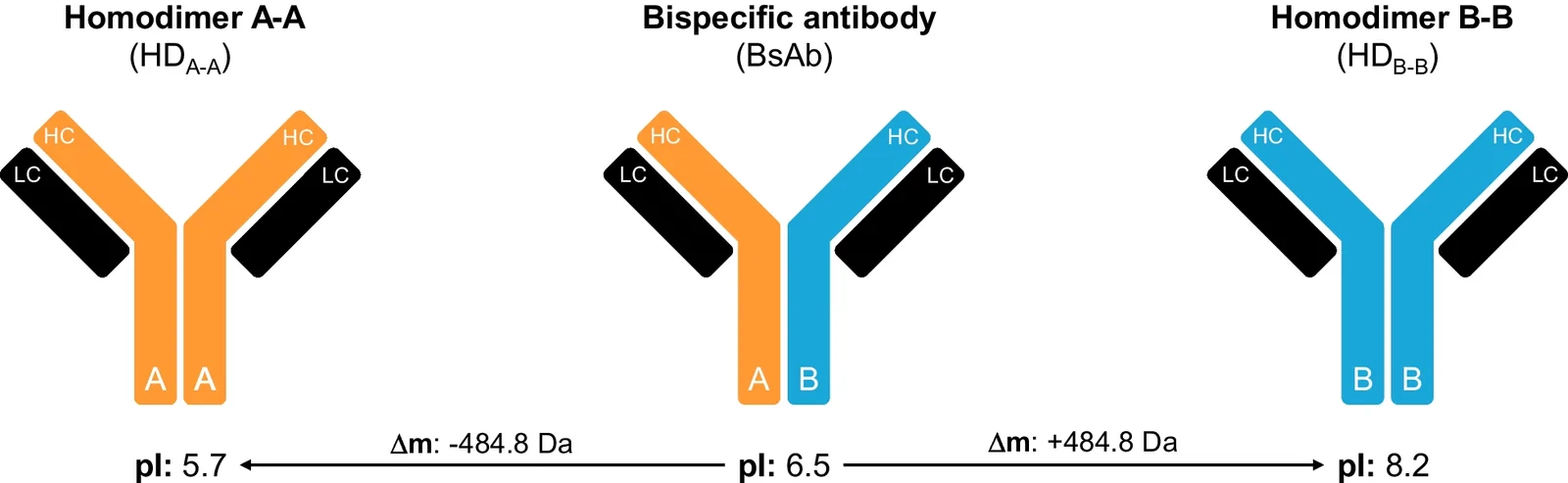
Schematic showing potential antibody variants formed by a cell line transfected with DNA encoding for the bispecific antibody (BsAb) used in this study. The correctly assembled BsAb features a common light chain (LC, black) and two different heavy chains: HC_A_ (orange) and HC_B_ (blue). The incorrectly assembled antibodies either contain only HC_A_ (homodimer HD_A-A_) or HC_B_ (homodimer HD_B-B_). The indicated isoelectric point (pI) was calculated based on the primary amino acid sequence using Expasy ProtParam. The mass difference (∆m, in Da) between the BsAb and the homodimers is indicated

In this study, we used an IgG-like BsAb as model protein, which comprises two distinct heavy chains (HC_A_ and HC_B_) and two light chains with identical amino acid sequence (LC). When the HCs and LCs are expressed by the cell, three potential antibodies could be formed (Fig. 1): the correctly assembled BsAb (comprised of LC-HC_A_-HC_B_-LC) or two HC homodimers comprised of LC-HC_A_-HC_A_-LC (HD_A-A_) or LC-HC_B_-HC_B_-LC (HD_B-B_). The BsAb and homodimers each have different pI and mass (Fig. 1), which makes MCE-MS an excellent technique to assess purity (homo- vs. heterodimer) and proteoform heterogeneity (charge variants) of this BsAb. Therefore, we developed a rapid workflow for both analytical purposes using a single MCE-MS platform.

### MCE-MS method development for BsAb analysis

**Fig. 2:**
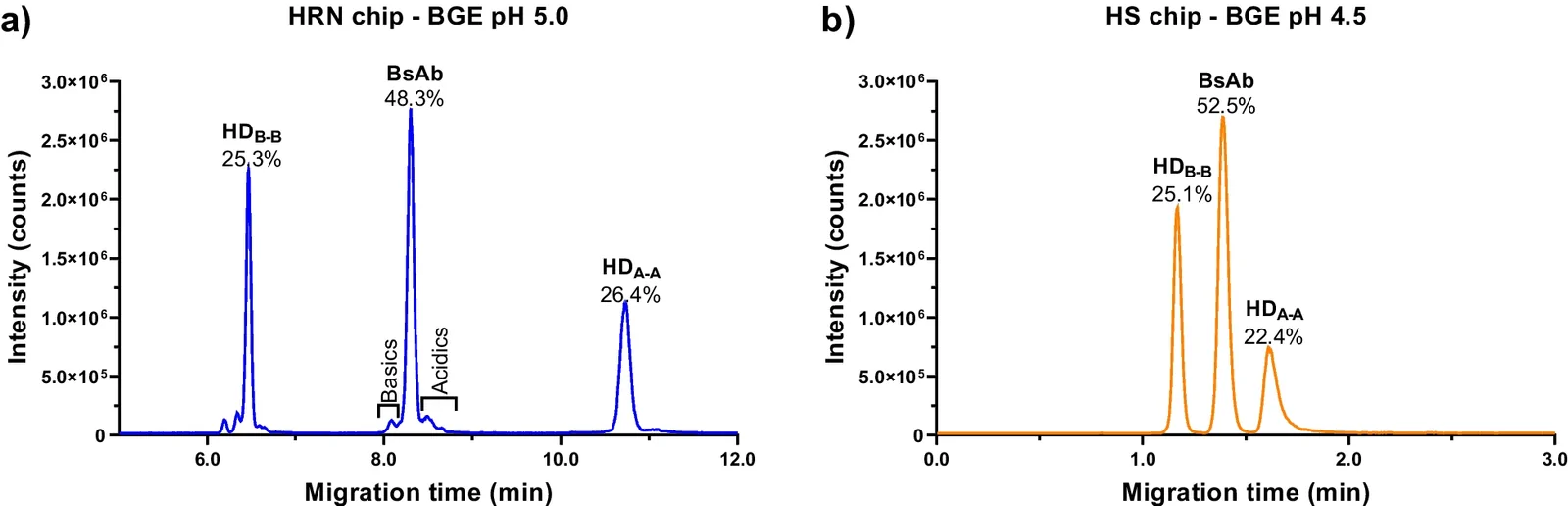
BPEs obtained by MCE-MS analysis of an HD_A-A_-BsAb-HD_B-B_ mixture (spiked ratio, 20:60:20) using **a** a 22-cm channel HRN chip and a BGE pH of 5.0 or **b** a 10-cm channel HS chip and a BGE pH of 4.5. The peaks of basic and acidic charge variants of the BsAb are indicated in **a**. Mass spectra of the observed peaks are depicted in Fig. S2. The measured fractional abundance of each Ab variant is denoted. Abbreviations used for peak assignment, see Fig. 1

As a starting point for an MCE-MS method for the determination of BsAb sample purity, experimental conditions previously described by our group and Wu et al*.* were applied [21, 22]. This entails the use of a High Resolution Native (HRN) chip, which features a 22-cm-length separation channel with a neutral coating based on a covalently bound polymer [23, 27]. A commercial BGE was employed containing ammonium acetate, isopropanol, and dimethyl sulfoxide (DMSO) at pH 5.5. A pure originator BsAb sample was spiked with HD_A-A_ and HD_B-B_, in a ratio of 20:60:20 (HD_A-A_-BsAb-HD_B-B_). The resulting base peak electropherogram (BPE) is shown in Fig. S1a, while the corresponding average mass spectra for each peak can be found in Figs. S2a, 2e, and 2i. Only two distinct high-abundant peaks were observed in the BPE (Fig. S1a) acquired using BGE pH 5.5. These peaks were attributed to the HD_B-B_ homodimer and BsAb, which migrated in descending order of their calculated pI (8.2 and 6.5, respectively), while the smaller peaks migrating before and after the main peak of the BsAb could be ascribed to basic and acidic charge variants of BsAb, respectively. No clear peak was observed for the HD_A-A_ homodimer. Since the pI of HD_A-A_ is very close to the pH of the BGE (5.7 vs. 5.5), the homodimer is expected to have a near-zero net charge, resulting in very low electrophoretic mobility. To increase the net charge and thus the electrophoretic mobility of the proteins, the BGE pH was lowered to 5.0 using acetic acid. The resulting BPE (Fig. 2a) indeed shows three clear Ab peaks with shorter migration times, including one for HD_A-A_ as the last migrating peak. Although the homodimer peaks exhibit different intensity and width, similar relative peak areas were attained (indicated in Fig. 2), as expected from spiking the sample with the same concentration of homodimers. When reducing the pH of the BGE further (pH 4.5), a distinct drop in resolution was observed, and acidic and basic variants of the BsAb signal could not be distinguished anymore (Fig. S1b). Therefore, in order to achieve separation of both the homodimers and the charge variants from the BsAb in a single run, a BGE of pH 5.0 was selected.

Notably, at pH 4.5, the HRN chip still provided sufficient resolution to separate the BsAb from its homodimers. Hence, when differentiation between the BsAb and the homodimers is the sole analytical requirement, the MCE-MS method can likely be accelerated by sacrificing resolution of the charge variants. In addition, incomplete or variable resolution of charge variants may complicate peak integration and thereby affect the relative quantification of the antibody species. To increase throughput, a chip with a short separation channel length of 10 cm was employed, while the field strength was doubled from 500 to 1000 V/cm (Fig. 2b). Under these conditions, three baseline-resolved peaks were obtained for the homodimers and the BsAb within less than 2 min, representing a five-fold reduction in run time. Furthermore, because the individual charge variants were intentionally no longer resolved, each antibody species produced a single well-defined peak, thereby improving confidence in peak integration and relative quantification. Again, both homodimers exhibited differences in peak intensity and shape, but their relative peak areas remained reasonably comparable. Potential differences in MS response factors among the antibody species obtained with this accelerated method are further discussed in section “BsAb and homodimer relative quantification”. Overall, the HS chip-based method demonstrated strong potential for rapid determination of BsAb purity and is henceforth referred to as high-throughput method. In contrast, when detailed profiling of BsAb charge variants is required, the HRN chip-based method employing a BGE at pH 5.0 can be applied; this method is hereafter referred to as the high-resolution method.

To ensure that low-abundant homodimers remain detectable in the high-throughput method, MS resolution was considered an important parameter to improve signal-to-noise (S/N). For Orbitrap-based mass analyzers, MS resolution is inversely proportional to the overall S/N; consequently lower MS resolution leads to increased sensitivity [9, 28]. Therefore, a low-resolution setting (15,000 at 200 *m/z*) was selected for the high-throughput method, as this approach prioritizes sensitivity for detecting low-abundance species, while the mass spectra are primarily used for confirmation of the identity of the BsAb and mispaired species. For the high-resolution MCE-MS method, higher MS resolution might be more beneficial; hence, a comparison of the 31+ charge state in the BsAb main peak of MS resolutions 15,000, 30,000 and 60,000 was carried out (Fig. S3). Reducing the resolution from 60,000 to 15,000 resulted in a 2.9-fold increase in S/N. However, this came at the expense of spectral resolution, as the full width at half maximum (FWHM) of the most abundant ion increased from 0.6 to 1.6 *m*/*z*, corresponding to an increase in deconvoluted peak width from 18.6 to 49.6 Da. To illustrate this effect, the peak pair highlighted in red in Fig. S3 (corresponding to the glycoforms G0F + G1F and G0F + G0F + GlcNAc) was unresolved at a resolution of 15,000 but could be distinguished at higher resolving powers. Hence, to strike a balance between signal intensity and sufficient resolving power, a resolution of 30,000 was selected for the high-resolution method.

### BsAb and homodimer relative quantification

**Fig. 3:**
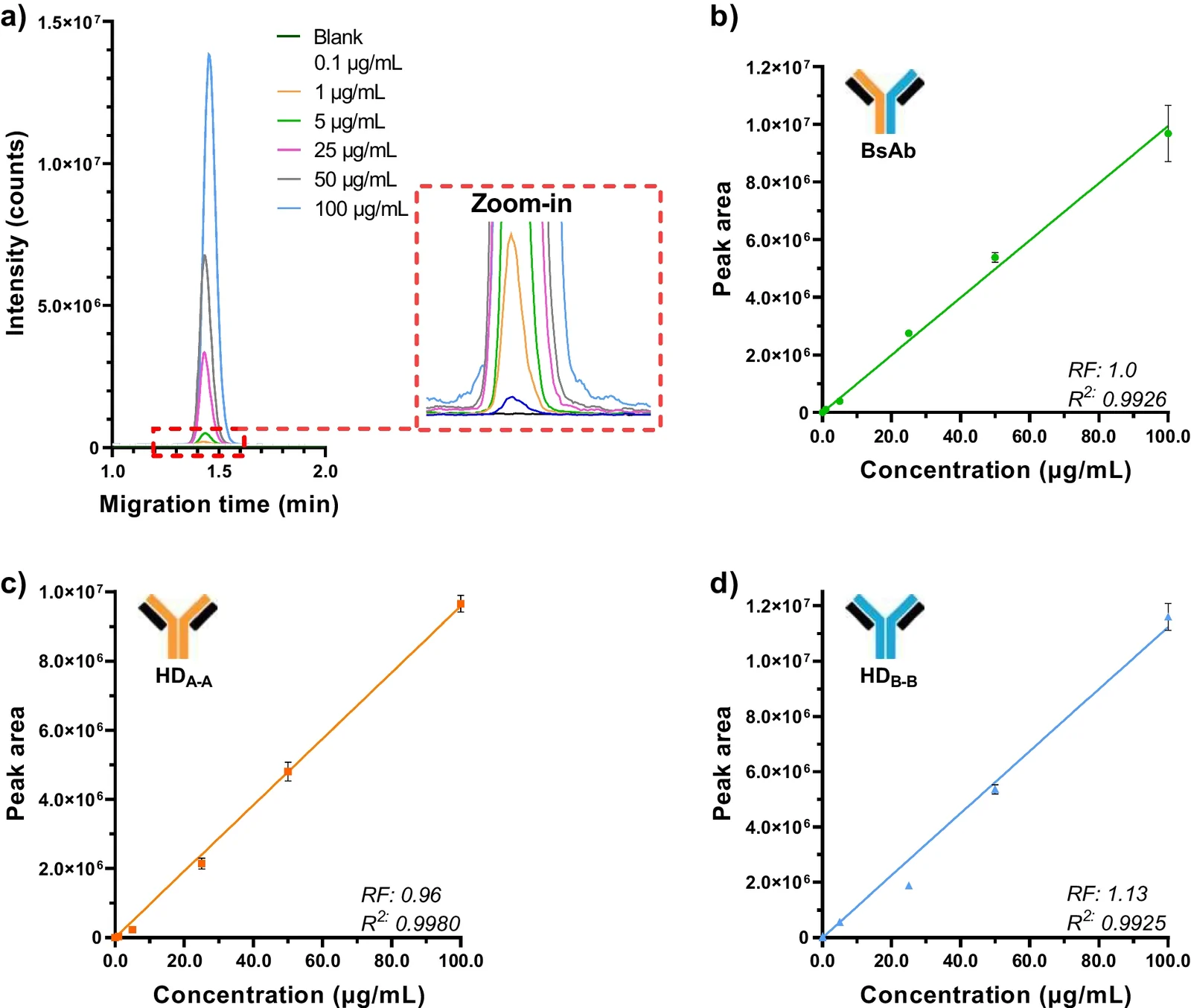
**a** Overlay and zoom-in of seven TIEs obtained during MCE-MS of a blank diluent and BsAb solutions with concentrations ranging from 0.1 to 100 µg/mL using the high-throughput MCE-MS method. **b**–**d** Calibration curves (peak area vs. injected concentration) based on the triplicate MCE-MS analysis of the different concentrations for **b** BsAb, **c** HD_A-A_, and **d** HD_B-B_. The response factor (RF) of the homodimers with respect to the BsAb (RF = 1.0), and the coefficient of determination (*R*^2^) are stated for each calibration curve

The BPE in Fig. 2b suggests that the homodimers and the BsAb exhibit different MS responses even though the homodimers were spiked at the same concentration. To investigate these potential differences in response and determine whether correction of the peak areas was required, calibration curves were constructed for each antibody species based on peak area versus injected concentration. Solutions containing the BsAb or homodimers at concentrations ranging from 0.1 to 100 µg/mL were analyzed in triplicate. The acquired total ion electropherograms (TIEs) for the BsAb are shown in Fig. 3a and demonstrate that the high-throughput method enabled detection of the BsAb across the entire tested concentration range. The resulting calibration curves (Fig. 3b–d) demonstrated excellent linearity for all Ab species, with high coefficients of determination (*R*^2^ > 0.99). The slopes of the calibration curves of the homodimers were compared with that of the BsAb to determine the response factors (RFs) for each variant, with the RF of the BsAb set to 1.0. HD_B-B_ showed a higher RF (1.13), whereas HD_A-A_ exhibited a slightly lower RF (0.96) compared to the BsAb. These differences suggest that, for this set of antibody species, MS response increased with increasing pI, which is consistent with theoretical expectations. The observed differences in response (Fig. 2b) can therefore be compensated for by applying the corresponding RFs to the measured peak areas.

**Fig. 4:**
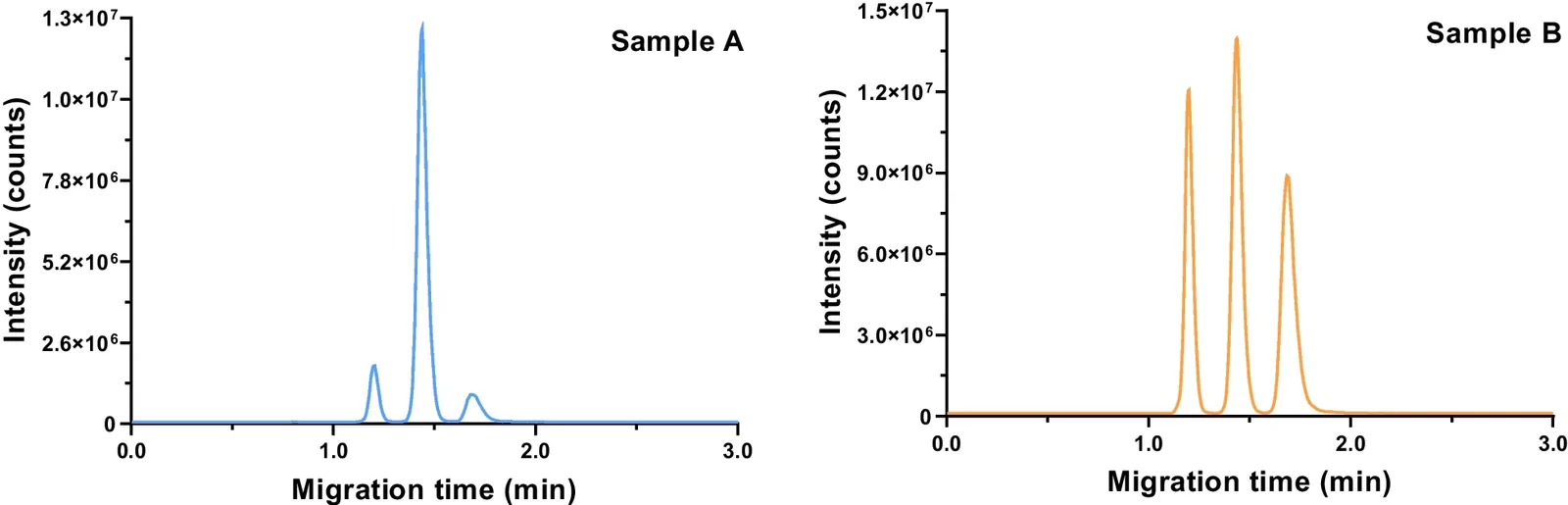
BPEs of HD_A-A_-BsAb-HD_B-B_ mixtures obtained using the high-throughput MCE-MS method. Spiked HD_A-A_-BsAb-HD_B-B_ ratio, 10:80:10 (sample A) and 25:50:25 (sample B)

The obtained RFs were subsequently used to determine the relative peak area of the BsAb and the homodimers present in two different spiked samples representative of actual BsAb samples. Samples A and B were prepared at HD_A-A_-BsAb-HD_B-B_ ratios of 10:80:10 and 25:50:25, respectively, and analyzed in triplicate using the high-throughput MCE-MS method. The resulting BPEs are depicted in Fig. 4, and the theoretical and calculated relative peak areas, together with the accuracy (calculated peak area divided by the theoretical peak area), are summarized in Table 1. All antibody species could be quantified with accuracy deviations from the theoretical values below 10%, except for HD_B-B_ in sample A, which showed a deviation of 16.9%. Furthermore, good repeatability was observed, with relative standard deviations (RSDs) ≤ 2.7% for all measured calculated peak areas. The migration times of the Ab variants were also highly reproducible, with RSDs below 1% for samples A and B individually, as well as when the two sample sets were considered together; when data from all concentration levels of the calibration curve were combined, the overall RSD remained ≤ 3.6% (Table S1). These performance characteristics indicate that the high-throughput MCE-MS method is well suited for screening large panels of clones and process development conditions, where rapid comparative assessment and ranking are prioritized.

**Table 1:** Theoretical and calculated peak area (%) ± 1 standard deviation of the homodimers and the BsAb, based on triplicate analysis of samples A and B (see Fig. 4) using the high-throughput MCE-MS method. The calculated peak areas were determined from the response factors based on the calibration curves shown in Fig. 3. The accuracy (%) indicates the calculated relative peak area with respect to the theoretical relative peak area

Sample	Theoretical peak area (%)	Calculated peak area (%)	Accuracy (%)
**HD**_**B-B**_			
A	10.0	8.3 ± 0.1	83.1 ± 1.5
B	25.0	26.8 ± 0.7	107.3 ± 3.0
**BsAb**			
A	80.0	82.4 ± 0.2	102.9 ± 0.2
B	50.0	46.0 ± 0.4	92.0 ± 0.7
**HD**_**A-A**_			
A	10.0	9.3 ± 0.1	93.3 ± 0.5
B	25.0	27.2 ± 0.7	108.8 ± 2.7

The calibration curves were established using protein A purified Ab species and are therefore directly applicable to samples with comparable matrix composition. However, previous studies have demonstrated that MCE-MS can also be applied to mAb samples in cell culture matrices without prior protein A purification [29, 30]. In such an approach, harvested cell culture supernatants are centrifuged and subsequently diluted prior to MCE-MS analysis, thereby eliminating the extra purification step. To evaluate whether this workflow was also suitable for the present application, the BPEs of a representative BsAb clone sample were compared after either protein A purification or after direct dilution of the cell culture supernatant (Fig. S4). In the diluted cell culture supernatant, two additional non-interfering peaks were observed with masses related to cysteinylated LC (+119 Da) and gluthathionylated LC (+305 Da) [20]. These components were absent in the protein A purified sample, as free LC species do not bind to Protein A and are therefore removed during purification. Compared with the purified sample, the BsAb peak in the diluted supernatant sample exhibited 2.2-fold reduction in signal intensity, likely due to ion suppression caused by the complex sample matrix containing salts, DNA, and host cell proteins. Consequently, additional correction factors were calculated for each Ab peak to compensate for matrix-dependent response differences (matrix factors, MFs), in combination with the RFs derived from the calibration curves (section S1). These MFs offer a means to correct for matrix effects caused by complicated matrices, such as diluted cell culture supernatants. Importantly, omitting the protein A purification step substantially reduces sample preparation time and associated costs, thereby further increasing the throughput of the method.

### Biosimilar BsAb clone screening

During the early stages of BsAb biosimilar development, numerous cell lines are transfected with DNA encoding the same amino acid sequence as the originator BsAb and cultured in parallel. Typically, cell lines that produce the most antibody are selected for further process development. Similar to mAbs, BsAb production titers are commonly determined using Protein A–based techniques, such as analytical Protein A chromatography or BLI with Protein A biosensors. However, these approaches measure the total concentration of antibody-related material, including mispaired Abs which also bind to Protein A. Consequently, clone selection based solely on total Ab titer may undesirably favor cell lines with high overall antibody production but lower BsAb purity [31].

**Fig. 5:**
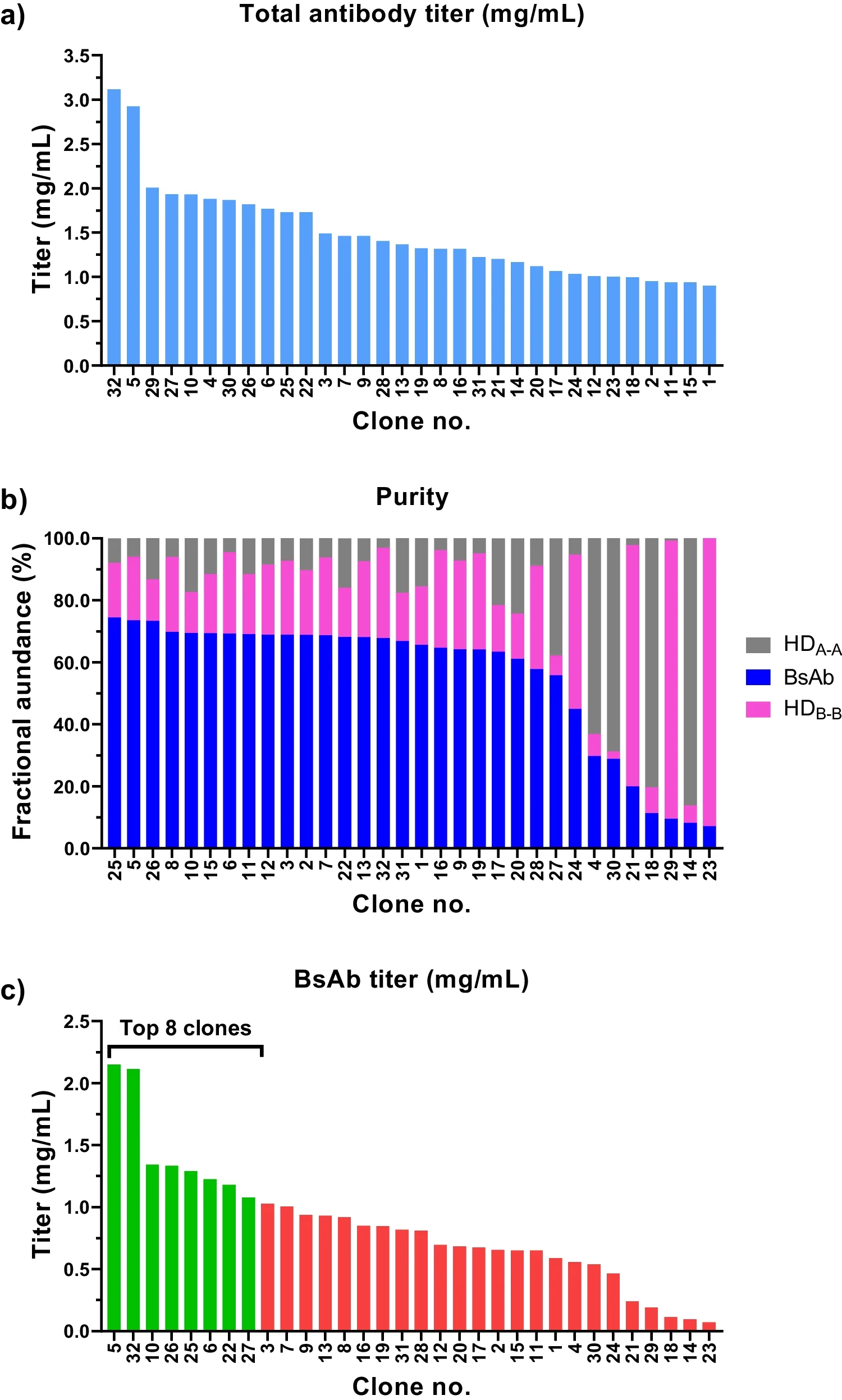
Bar charts depicting **a** the total antibody titer (mg/mL) and **b** the fractional abundance (%) of the BsAb and the homodimer variants as obtained with the high-throughput MCE-MS method, and **c** the resulting BsAb titer (mg/mL) of the 32 clone BsAbs

To evaluate the impact of BsAb purity on clone selection, the high-throughput MCE-MS method was applied to 32 clonal cell lines transfected with DNA encoding for the same BsAb biosimilar. Following harvest, centrifugation, and titer determination, the cell culture supernatants were diluted and directly analyzed by MCE-MS. The total Ab titers determined by BLI are depicted in Fig. 5a, showing titers ranging from 0.9 mg/mL (clone 1) to 3.1 mg/mL (clone 32). In contrast, the purity-based clone ranking obtained using the high-throughput method (Fig. 5b) revealed substantial variation in BsAb fractional abundance, ranging from 7.1% (clone 23) to 74.5% (clone 25), indicating that homodimer formation differed considerably among clones. Subsequently, the BsAb purity of each clone was used to calculate the BsAb titer, resulting in the ranking shown in Fig. 5c. Notably, the highest BsAb-producing clones were not necessarily those with the highest total antibody titer, highlighting the importance of incorporating purity assessment during clone selection. For instance, clone 29 exhibited the third highest total antibody titer but showed a BsAb purity of only 9.5%. Consequently, this clone was not selected based on BsAb titer, despite being among the highest-producing clones according to conventional titer determination alone. Similar cases were identified for two other clones (nos. 4 and 30). These findings demonstrate that the high-throughput MCE-MS method can improve BsAb clone selection based on BsAb purity assessment.

To further refine clone selection, the quality attributes of the highest BsAb-producing cell lines were evaluated using the high-resolution MCE-MS method. Hence, the top eight clone BsAbs based on BsAb titer of this case study (highlighted in green in Fig. 5c), together with the originator BsAb, were analyzed for CQAs such as charge variants and glycosylation profile using the high-resolution MCE-MS method. Importantly, the samples prepared for purity assessment remained in the autosampler, and only the chip and BGE were exchanged prior to analysis, requiring approximately 30 min to change from the high-throughput to the high-resolution setup. The resulting BPEs can be found in Fig. S5.

**Fig. 6:**
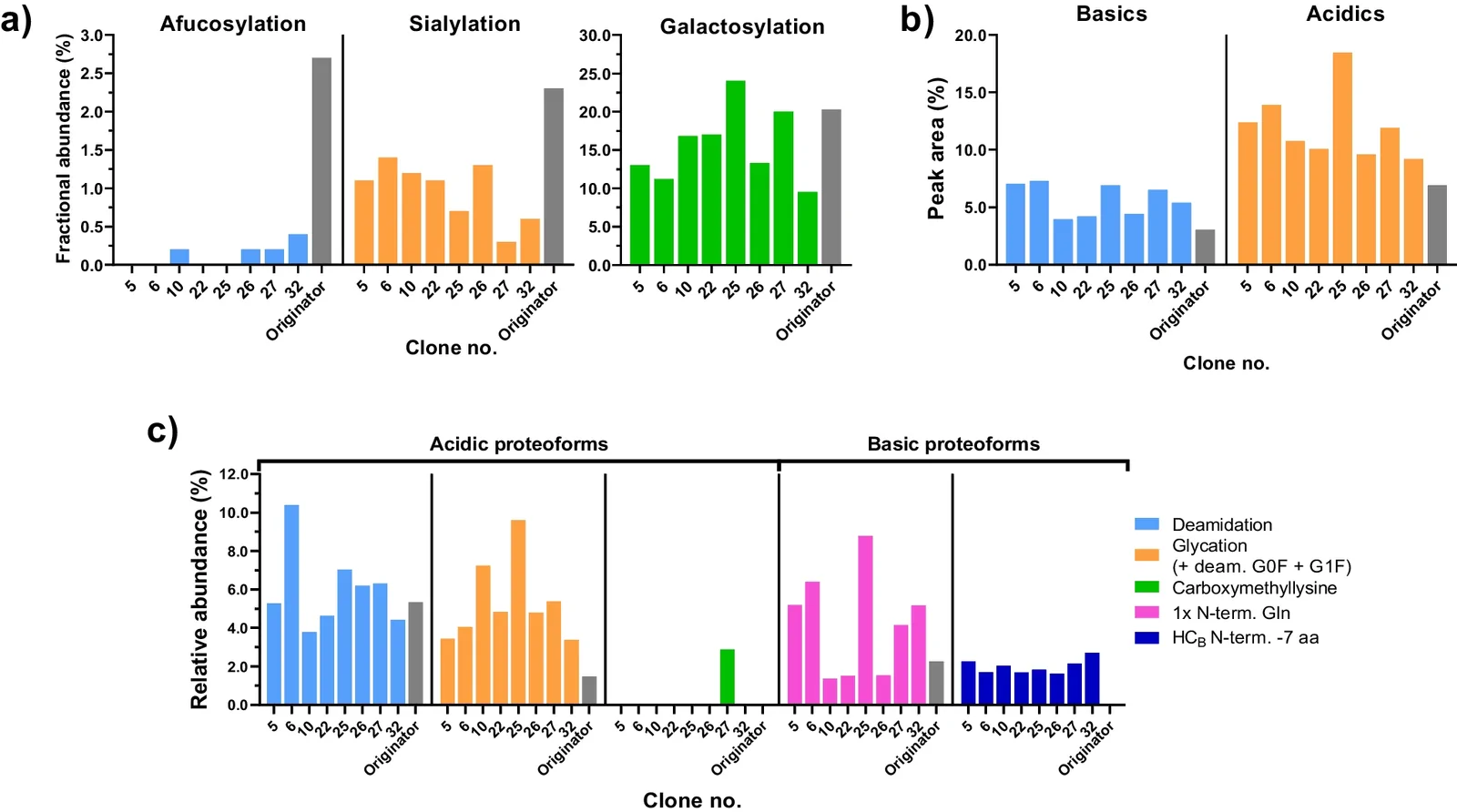
Bar charts depicting **a** the relative levels (%) of afucosylation, sialylation, and galactosylation; **b** the peak area (%) of the basic and acidic charge variants; and **c** the relative abundance of the identified acidic and basic-migrating proteoforms of the selected top 8 clones and originator BsAb, obtained with the high-resolution MCE-MS method. The acidic-migrating proteoforms were identified as asparagine deamidation and/or isomerization, glycation (and the deamidated glycoform G0F + G1F) and a single carboxymethyllysine. The basic-migrating proteoforms were identified as a single N-terminal glutamine and the truncation of the first seven amino acids on heavy chain B (HC_B_)

Figure 6 summarizes the results obtained after sliding-window deconvolution of the mass spectra corresponding to the BsAb and its charge variants. First, the glycoforms of the clones and originator BsAb were assessed. The fractional abundances of the glycoforms (Table S2) were used to calculate the levels of afucosylation (absence of core fucose in glycoforms), sialylation, and galactosylation, which are depicted in Fig. 6a. These glycosylation indicators were chosen as they influence the BSAb effector functions such as antibody-dependent cell-mediated cytotoxicity (ADCC) and complement-dependent cytotoxicity (CDC) [32]. Overall, most clone-derived BsAbs differed substantially from the originator, indicating that further optimization of media and feed conditions would likely be required to obtain a more comparable glycosylation profile. The clone samples were additionally compared based on the relative peak area of the charge variants and their associating charge-migrating proteoforms (Fig. 6b, c). The electropherograms of all clone-derived BsAbs (Fig. S5) exhibited two basic charge-variant peaks, whereas only a single basic peak was observed for the originator BsAb. The basic peak closest to the main peak displayed a mass shift of + 17 Da for all samples including the originator, which was attributed to incomplete conversion of an N-terminal glutamate residue to pyroglutamic acid based on mass shift and migration behavior. The second basic variant, absent in the originator BsAb, exhibited a mass decrease of −766 Da relative to the main proteoform which could be explained by an N-terminal truncation involving the first seven amino acids of HC_B_. This truncation resulted in a net gain of two charges relative to the main BsAb proteoform, causing the truncated species to migrate ahead of the first basic variant peak.

With respect to the acidic charge variants, three distinct types of acidic-migrating proteoforms were observed in addition to the sialylated glycoforms. Proteoforms exhibiting a small mass increase (+ 1–5 Da) were detected in the acidic regions of all samples. These minor mass shifts in combination with their longer migration times fit well with one or more asparagine deamidations and/or aspartic acid isomerizations, which are commonly occurring PTMs in antibodies [33, 34]. Sliding-window deconvolution algorithms commonly struggle to accurately quantify deamidated charge variants because these species are merged with their non-deamidated counterparts due to their highly similar masses. By applying the Peak Picker function of the ProSight Native software, separate extracted ion electropherograms (EIEs) were generated for these masses based on the peaks detected in the BPE. This enabled more accurate quantification of deamidation-related proteoforms since they migrate as an acidic charge variant. An example is shown in Fig. S6. We also observed a +162 Da mass shift in the acidic region relative to the main proteoform. This mass shift was attributed either to glycation or to the deamidated variant of the G0F + G1F glycoform combination, as both modifications are expected to migrate as acidic variants and exhibit highly similar masses. Although these two possibilities could not be distinguished in the present analysis, both modifications are undesirable and should therefore be monitored and controlled. To determine the actual degree of glycation, deglycosylation of the BsAb would be needed. Furthermore, clone 27 exhibited an additional proteoform with a mass shift of + 58 Da, which can most likely be identified as carboxymethyllysine, an advanced glycation end product (AGE).

Based on the outcomes above, clone selection was performed according to a previously reported scoring procedure [21]. In addition to the quality attributes discussed above, BsAb purity was incorporated into the scoring strategy. The resulting clone selection ranking (Fig. S7) showed relatively small differences among the top-performing clones, with clones 10, 22, and 26 obtaining the highest overall scores. Altogether, this clone screening case study demonstrates that the developed MCE-MS workflows enable rapid and information-rich assessment of both BsAb purity and CQAs using a single, straightforward sample preparation procedure.

### BsAb downstream purification process evaluation

Downstream processing (DSP) of BsAbs from harvested cell culture requires careful selection and optimization of purification strategies to obtain a high-quality end product while minimizing impurities such as homodimers and host cell proteins (HCPs). Typically, multiple purification techniques, including filtration and chromatography, are applied sequentially to selectively capture and purify the BsAb [35]. Each purification step involves process parameters that require optimization to ensure that the highest product quality and yield are obtained. The resulting process intermediate samples from the various steps need to be analyzed to guide the selection of optimal process conditions. To accelerate this decision-making process, the developed MCE-MS workflow was evaluated by monitoring a representative BsAb DSP pilot procedure that was set up for this study, with particular focus on tracking homodimer removal throughout the purification sequence.

The pilot procedure consisted of a Protein A chromatography step for capturing all Ab species, followed by an anion exchange chromatography (AEX) step for the removal of HCPs and DNA. AEX was operated in flow-through mode, which means that the BsAb was expected to not bind to the stationary phase, whereas HCPs and DNA were retained [35]. AEX was followed by a cation exchange chromatography (CEX) step, of which the eluate was collected in six fractions (F1 to F6, see Fig. S8). Fractions were pooled based on their BsAb purity. The resulting CEX pool was subjected to ultrafiltration/diafiltration (UFDF) for cleanup and buffer exchange into the formulation buffer, followed by sterile filtration and final dilution to obtain the drug substance (DS).

**Fig. 7:**
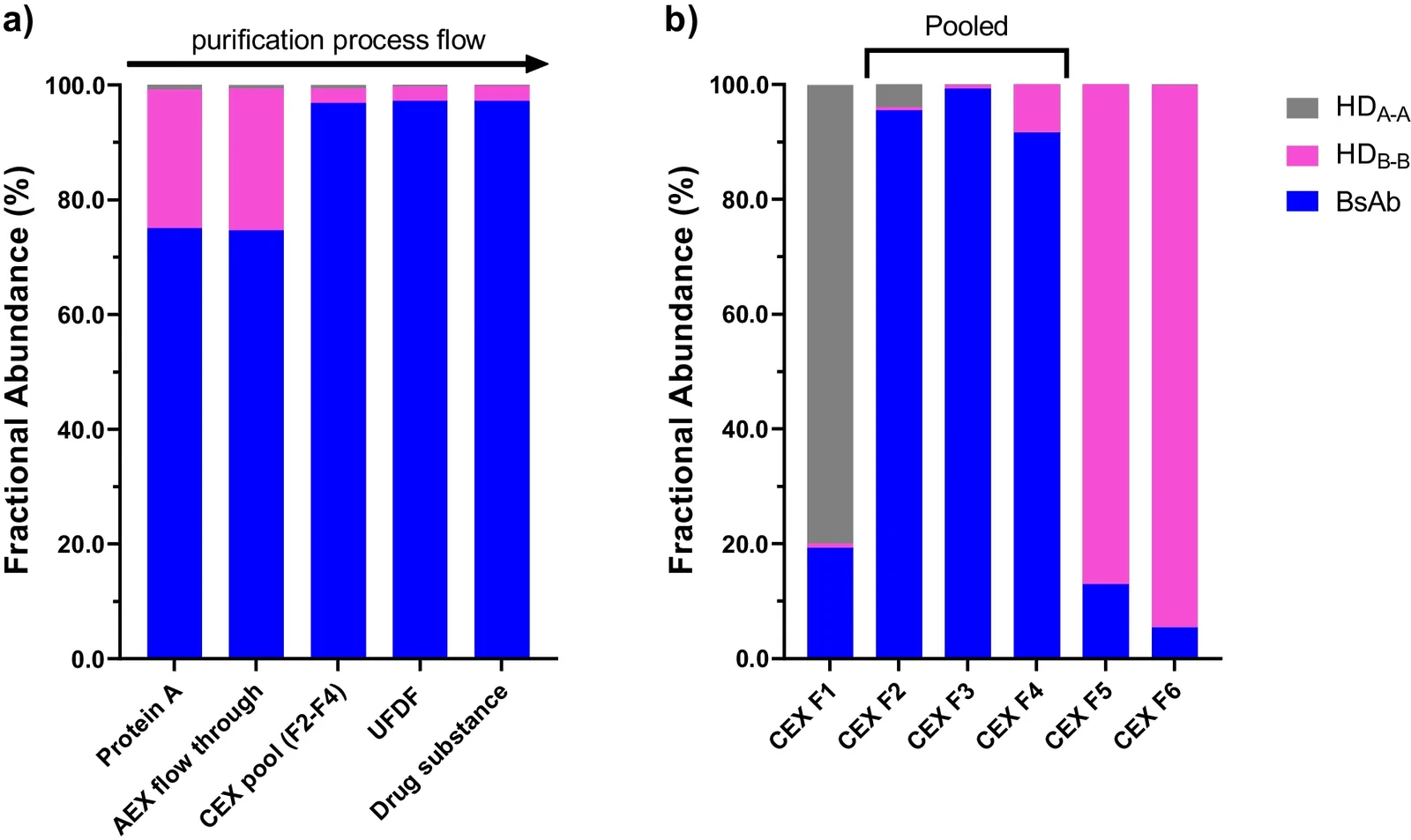
Bar charts depicting the fractional abundance (%) of the BsAb and the homodimers for **a** stages of the downstream purification process and **b** the separate CEX fractions (as indicated in Fig. S8) as determined by the high-throughput MCE-MS method. The arrow in **a** denotes the successive purification steps. The bracket in **b** indicates which fractions were pooled for further purification

The BsAb purity determined after each purification step using the high-throughput MCE-MS method is shown in Fig. 7a, while the corresponding BPEs are presented in Fig. S9. After protein A purification, the sample exhibited a BsAb purity of 75.0%. Comparable purity was observed after the AEX stage, indicating that this step did not significantly improve BSAb purity. The UV chromatogram of the preparative CEX separation, including the collected fractions, is shown in Fig. S8. As expected, the BPEs (Fig. S9) and the fractional abundances of the Ab variants (Fig. 7b) indicate that the elution order in CEX is reversed compared to MCE; the low pI HD_A-A_ elutes first, followed by the BsAb and HD_B-B_. The calculated BsAb purities of the individual CEX fractions (Fig. 7b) showed that fractions F2–F4 contained the highest BsAb purities. Although fraction F3 exhibited the highest purity (99.2%) compared with fractions F2 and F4 (95.5% and 91.6%, respectively), fractions F2–F4 were pooled to ensure sufficient product recovery from the CEX process. Following pooling, the BsAb purity increases to around 97%, which was maintained after UFDF and DS formulation steps. These results indicate that the CEX step was particularly effective for homodimer removal, although further process optimization would still be required to achieve > 99% BsAb purity.

**Fig. 8:**
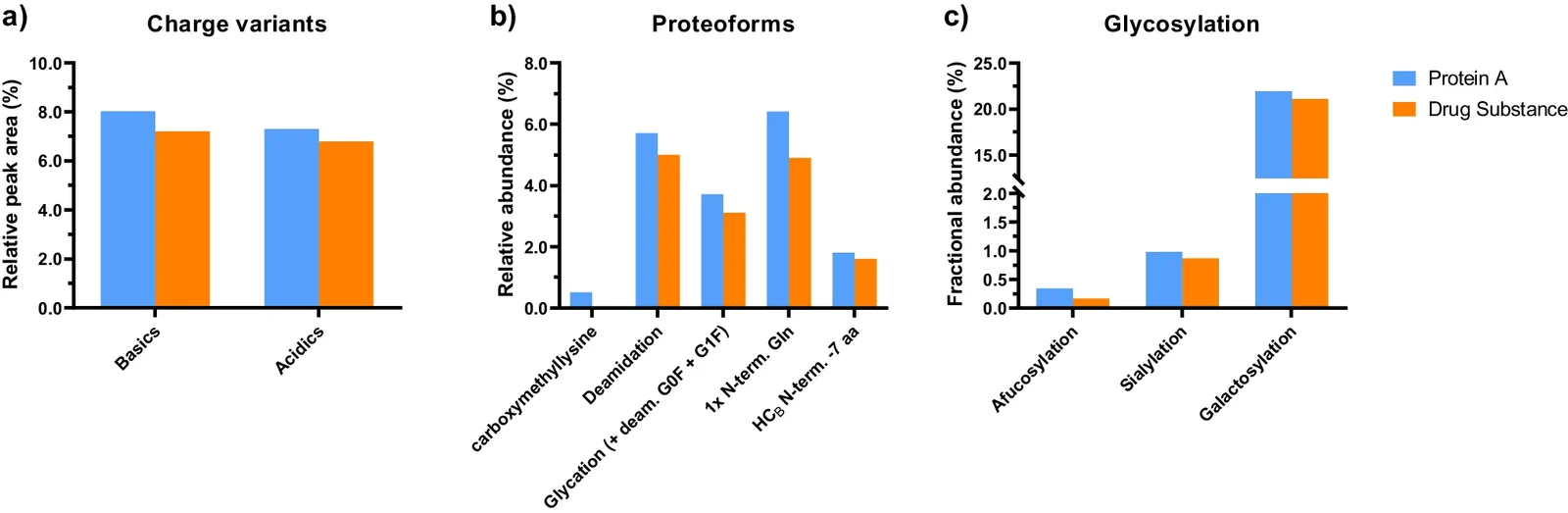
Bar graphs showing the **a** peak area (%) of the basic and acidic charge variants, **b** the relative abundance (%) of the charge-migrating proteoforms, and **c** the levels (%) of afucosylation, sialylation, and galactosylation, obtained with the high-resolution MCE-MS method for the start (after Protein A chromatography, in blue) and end sample (after formulation into drug substance, in orange) of the downstream purification process

While the high-throughput MCE-MS method provides important insights into the change in BsAb impurities throughout DSP, it is equally important to evaluate whether the purification process affects the quality profile of the BsAb itself. For example, the low-pH elution conditions of Protein A chromatography in combination with a viral inactivation step may induce modifications such as antibody aggregation, succinimide formation, and aspartic acid isomerization [36, 37]. To investigate whether the purification altered the BsAb quality profile, the start (after Protein A chromatography) and end sample (DS) were analyzed using the high-resolution MCE-MS method. The resulting glycosylation and charge-variant profiles are depicted in Fig. 8. Interestingly, the relative abundance of charge-variant peaks and their associated charge-inducing proteoform levels were all reduced for the DS sample compared to the start sample. These findings indicate that the pilot DSP process investigated here did not induce problematic charge variants, such as deamidation or glycation, but instead reduced their relative abundance. The levels of glycosylation-related attributes were also slightly lower in the DS sample, although these differences were minor (≤ 0.8%). Overall, this pilot study demonstrates how the high-throughput method can rapidly assess BsAb purity in DSP process intermediates, thereby enabling efficient monitoring of purification performance and facilitating process optimization when purity specifications are not achieved. In parallel, the high-resolution method can be applied to study the effect of process parameters on the BsAb quality profile. For instance, different preparative IEX buffer systems in DSP could first be screened for their effectiveness in removing mispaired antibody species using the high-throughput method, after which selected conditions could be evaluated in greater detail using the high-resolution workflow.

The MCE-MS workflow described in this study is expected to be applicable to different BsAb molecules with different formats. To optimize the separation of antibody variants for other BsAbs, the selectivity of the method can be readily tuned by adjusting the BGE pH, thus requiring only limited method development. In this work, we demonstrated that a pI difference of 0.8 between antibody variants results in baseline-resolved separation with the high-throughput method, although smaller differences may also be resolved. For instance, we were also able to separate the deamidated BsAb from the parent BsAb using the high-resolution method, which only has a calculated pI difference of approximately 0.08. For very complex BsAb formats with smaller pI differences or a larger number of possible variants, the high-resolution method may be required to achieve desired separation.

## Conclusion

In this work, a workflow consisting of two easily interchangeable MCE-MS methods was developed for the evaluation of BsAb purity and quality attributes. The high-throughput method enabled separation of the homodimers from the BsAb in just 3-min run time, allowing efficient purity assessment for large BsAb sample sets. Differences in MS response between the antibody species were systematically evaluated and corrected for using response factors, resulting in more accurate determination of BsAb purity. In addition to separating the homodimers from the BsAb, the high-resolution method enabled characterization of BsAb charge variants and associated proteoforms, thereby facilitating assessment of CQAs, including glycosylation profiles and charge-related modifications. Both methods were also successfully applied to diluted harvested cell culture supernatants, eliminating the need for a time-consuming protein A purification step prior to analysis. Together, the developed methods constitute a versatile analytical workflow in which large sample sets can first be rapidly screened for BsAb purity using the high-throughput method, followed by in-depth characterization of selected samples using the high-resolution workflow. As demonstrated in the presented case studies, these methods have potential utility across multiple stages of BsAb development and manufacturing, where they can support faster and more informed decision-making based on both BsAb purity and product quality attributes.

## Supplementary Information

Below is the link to the electronic supplementary material.

Supplementary file1 (PDF 804 KB)

## Data Availability

The data used for this study cannot be shared due to confidentiality concerning the identity of the BsAb biosimilar samples.
