# Supplementary material for: Rapid purity and charge-variant analysis of bispecific antibodies using microfluidic capillary electrophoresis-mass spectrometry
Source: Anal Bioanal Chem. 2026 Aug 22;418(22):7285–99. doi: 10.1007/s00216-026-06748-z (PMC13736329; doi:10.1007/s00216-026-06748-z)
Supplement: Supplementary file 1 [file 216_2026_6748_MOESM1_ESM.pdf]

Supporting information

Rapid purity and charge-variant analysis of bispecific antibodies using microfluidic capillary electrophoresis-mass spectrometry

Ruben Cageling[a,b,c], Karin Lubbers-Geuijen[a], Govert W. Somsen[b,c] and Kevin Jooß[b,c*]

[a] Protein Purification & Characterization department, Polpharma Biologics Utrecht, Yalelaan 46, 3584 CM, Utrecht, The Netherlands

[b] Division of BioAnalytical Chemistry, Department of Chemistry and Pharmaceutical Sciences, Amsterdam Institute for Molecular and Life Sciences (AIMMS), Vrije Universiteit Amsterdam, de Boelelaan 1085, 1081 HV Amsterdam, The Netherlands

[c] Centre for Analytical Sciences Amsterdam (CASA), Amsterdam, The Netherlands

*Corresponding author: Kevin Jooß, address: Division of BioAnalytical Chemistry, Department of Chemistry and Pharmaceutical Sciences, Amsterdam Institute for Molecular and Life Sciences (AIMMS), Vrije Universiteit Amsterdam, de Boelelaan 1085, 1081 HV Amsterdam, The Netherlands; email address: k.jooss@vu.nl

S1. Response and matrix correction factors

Response Factors (RFs)

As demonstrated in section 3.2, the calibration curves revealed that the homodimers and the BsAb give different MS responses for the same injected concentrations. To adjust for the difference in MS response, response factors (RFs) were calculated based on the slope of calibration curve for each homodimer variant compared to the slope of the BsAb (RF_{BsAb} = 1.0). The formula is similar for $HD_{A\text{-}A}$ and $HD_{B\text{-}B}$ and is described in Eq. 1 for $HD_{A\text{-}A}$:

$$RF_{HD_{A-A}} = \frac{Slope_{HD_{A-A}}}{Slope_{BsAb}} \qquad \text{(Eq. 1)}$$

Next, the RFs are used to adjust the obtained MS peak area after peak integration of the TIE using Eq. 2:

$$A_{HD_{A-A,adj}} = \frac{A_{HD_{A-A}}}{RF_{HD_{A-A}}} \qquad \text{(Eq. 2)}$$

With $A_{HD_{A-A}}$ being the MS peak area of $HD_{A\text{-}A}$ obtained after integration and $A_{HD_{A-A,adj}}$ the adjusted MS peak area after correction with the RF. The relative MS peak area ($A\%$) based on the adjusted peak area was determined as follows (Eq. 3):

$$A\%_{HD_{A-A},adj} = \frac{A_{HD_{A-A,adj}}}{A_{HD_{A-A,adj}} + A_{HD_{B-B,adj}} + A_{BsAb}} \qquad \text{(Eq. 3)}$$

Matrix Factors (MFs)

If a sample is in a different matrix as the calibration curve, then a matrix correction should be performed by applying matrix factors (MFs). For this, a sample was run in the non-purified and Protein-A purified matrix. The relative MS peak areas after integration were used to determine the MFs (Eq. 4):

$$MF_{HD_{A-A}} = \frac{A\%_{HD_{A-A,crude}}}{A\%_{HD_{A-A,pure}}} \qquad \text{(Eq. 4)}$$

Subsequently, the RFs were adjusted ($RF_{HD_{A-A,adj}}$) with the MF to correct for the matrix effect, as shown in (Eq. 5).

$$RF_{HD_{A-A,adj}} = \frac{RF_{HD_{A-A}}}{MF_{HD_{A-A}}} \qquad \text{(Eq. 5)}$$

The adjusted RFs can then be applied as described in (Eq. 2) and (Eq. 3).

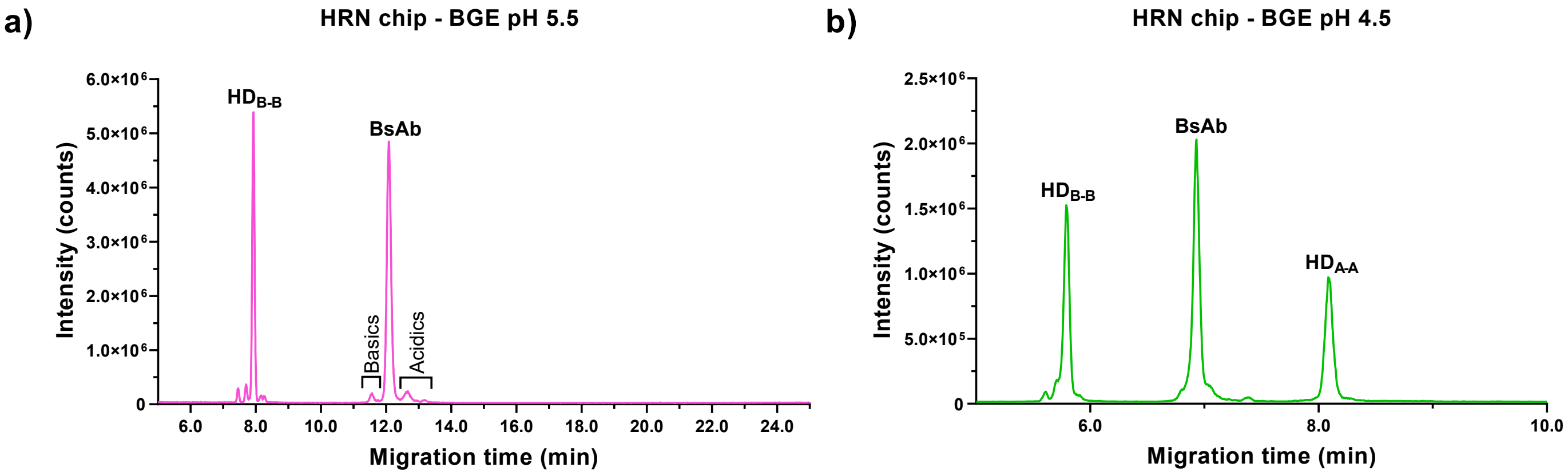

Fig. S1 BPEs obtained by MCE-MS analysis of an $HD_{A\text{-}A}$-BsAb-$HD_{B\text{-}B}$ mixture (spiked ratio, 20:60:20) using a) a 22-cm channel HRN chip and a BGE pH of 5.5 or b) a BGE pH of 4.5. The peaks of basic and acidic charge variants of the BsAb are indicated in a). Mass spectra of the observed peaks are depicted in Fig. S2. The measured fractional abundance of each Ab variant is denoted. Abbreviations used for peak assignment, see Fig. 1

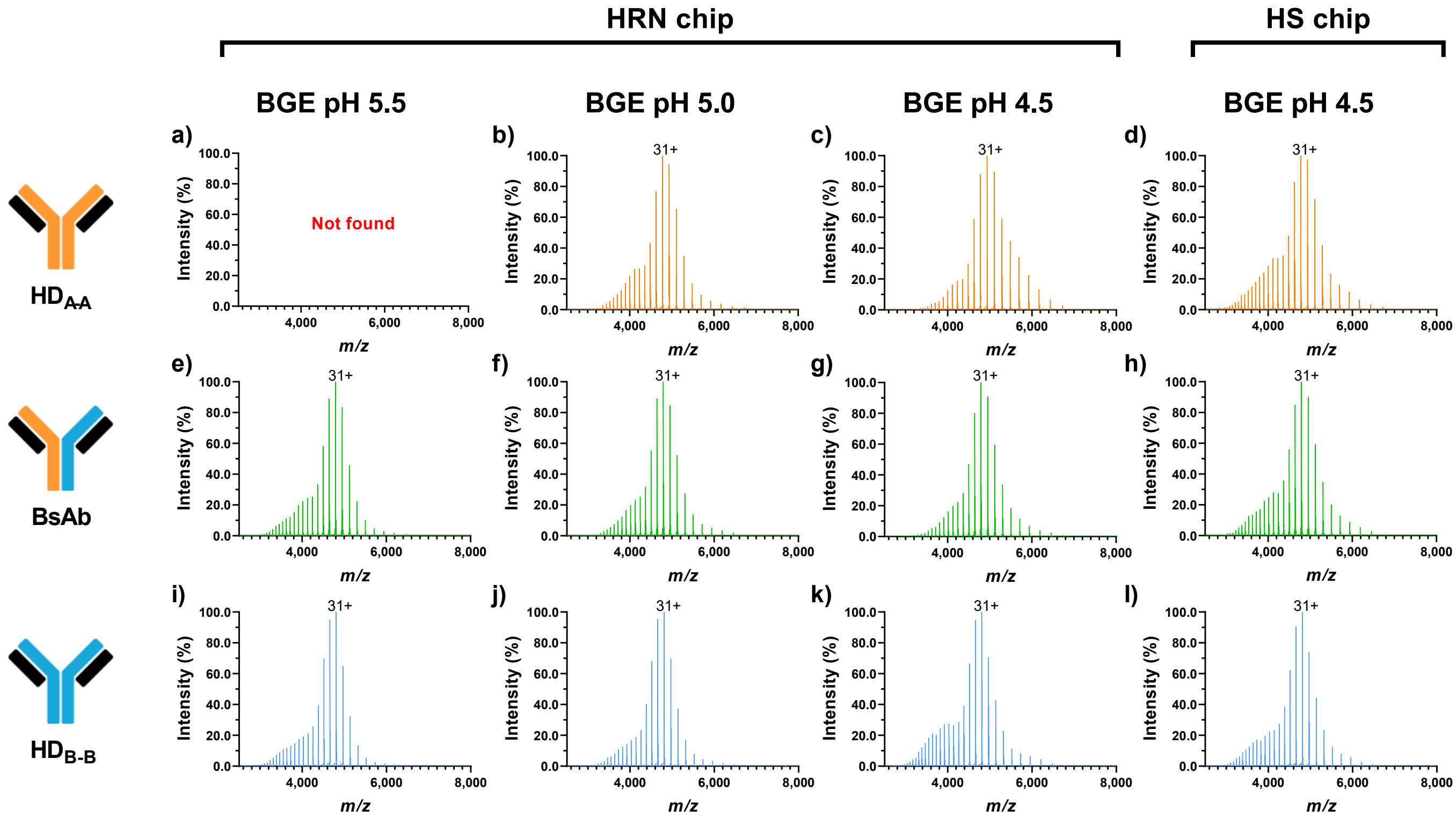

Fig. S2 Normalized mass spectra of the main peaks assigned to the antibody variants HD_{A-A} (a-d), BsAb (e-h), and HD_{B-B} (i-l), obtained by MCE-MS analysis of a spiked BsAb sample. Panels a, e, and i were recorded using an HRN chip with BGE at pH 5.5; panels b, f, and j with BGE at pH 5.0; and panels c, g, and k with BGE at pH 4.5. Panels d, h, and l were recorded for the same sample using an HS chip with CVA BGE at pH 4.5. No mass spectrum corresponding to HD_{A-A} was observed for the HRN chip with BGE at pH 5.5 (panel a). The 31+ charge state is indicated in each spectrum

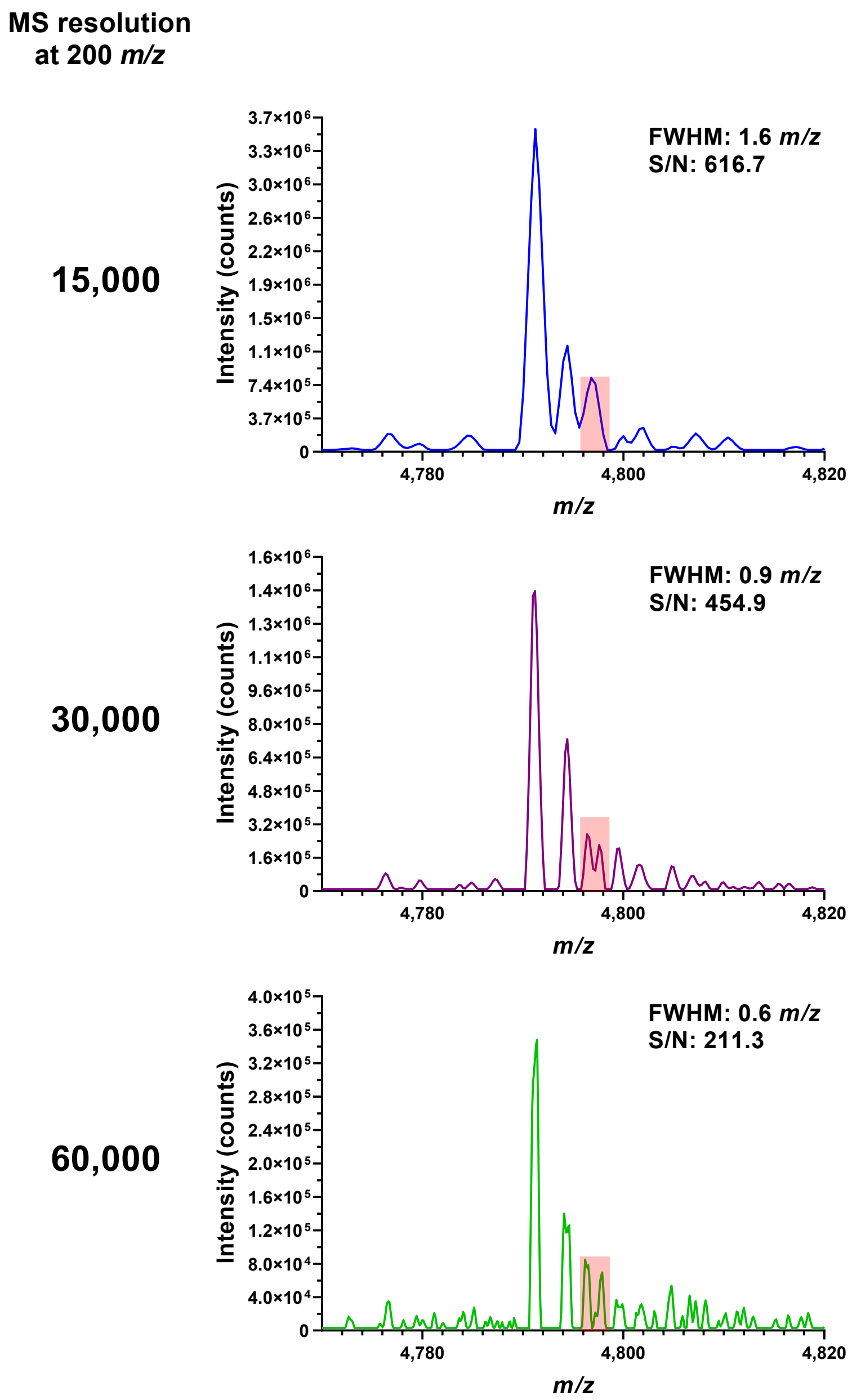

Fig. S3 Mass spectra obtained at the peak apex of the man BsAb peak, showing the most abundant charge state (31+). All mass spectra were acquired with MCE-MS using an HRN chip and BGE pH 4.5, applying an MS resolution at 200 *m/z* of either 15,000 (top panel), 30,000 (middle panel) or 60,000 (bottom panel), respectively. The signal-to-noise ratio (S/N) and the full width half maximum (FWHM) for the most intense *m/z* peak is denoted in each mass spectrum

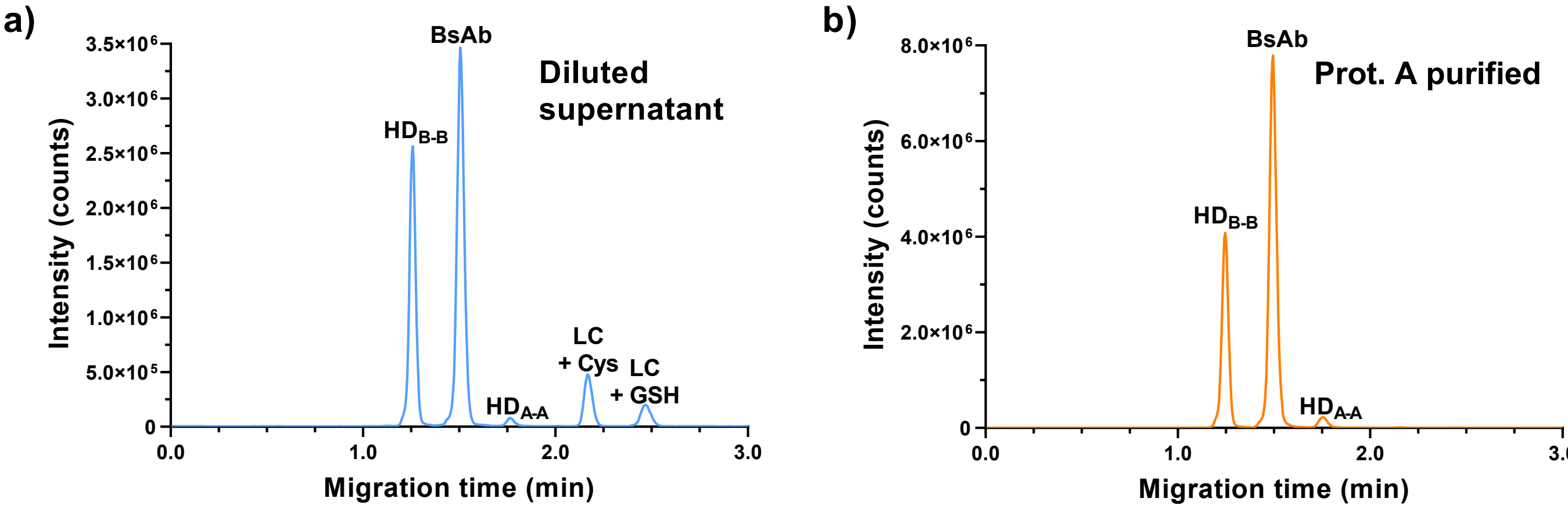

Fig. S4 BPEs of clone 32 sample measured with the high-throughput MCE-MS method by a) directly diluting and injecting crude cell culture supernatant or b) after protein A purification. The two additional peaks in the crude sample are a cysteinylated light chain (LC + Cys) and a glutathionylated light chain (LC + GSH) which do not bind to protein A and are therefore not present in the purified sample

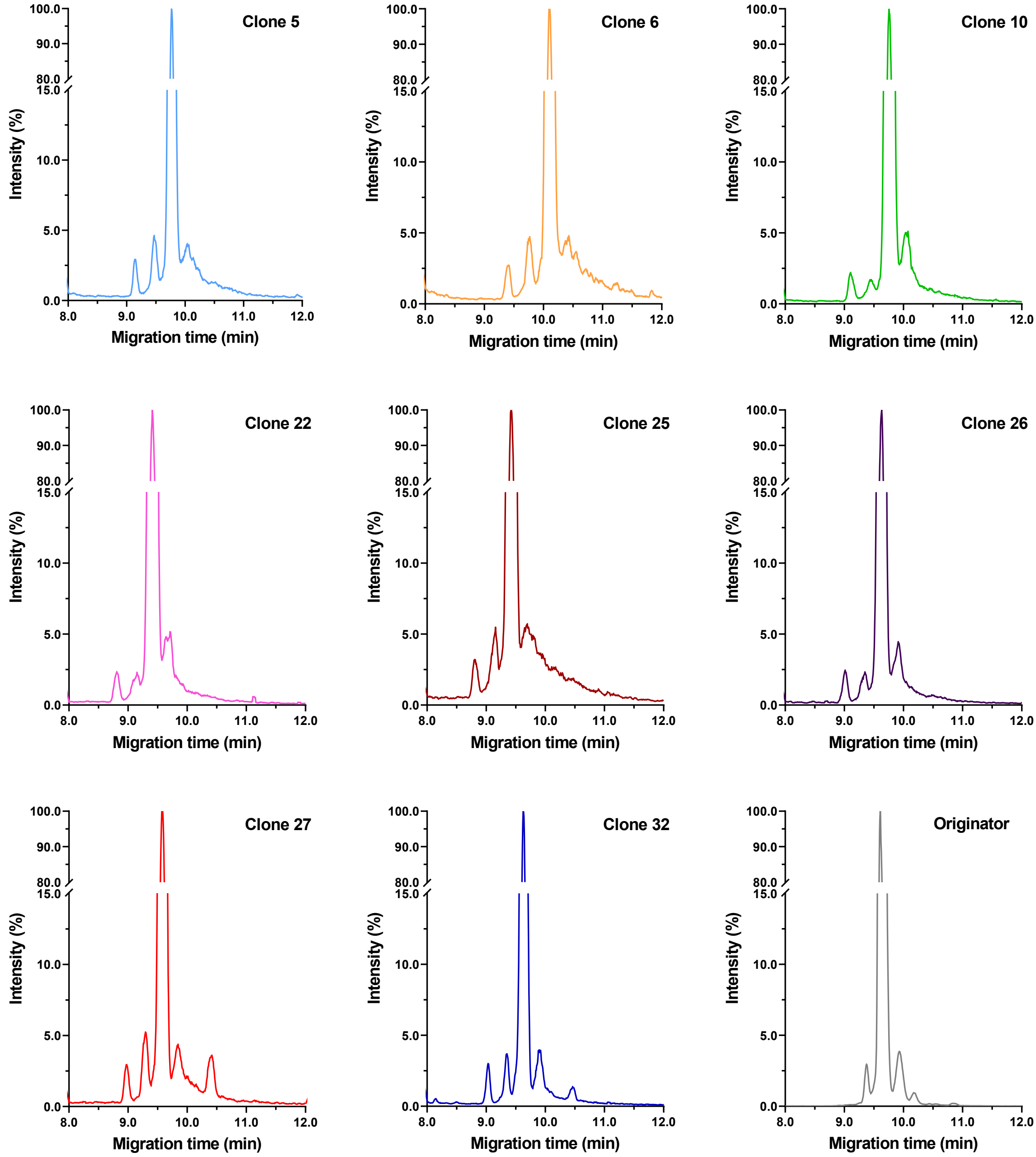

Fig. S5 BPEs depicting the selected top 8 clones and originator BsAb, obtained by the high-resolution MCE-MS method. The migration time window is focused on the BsAb main peak and its charge variants

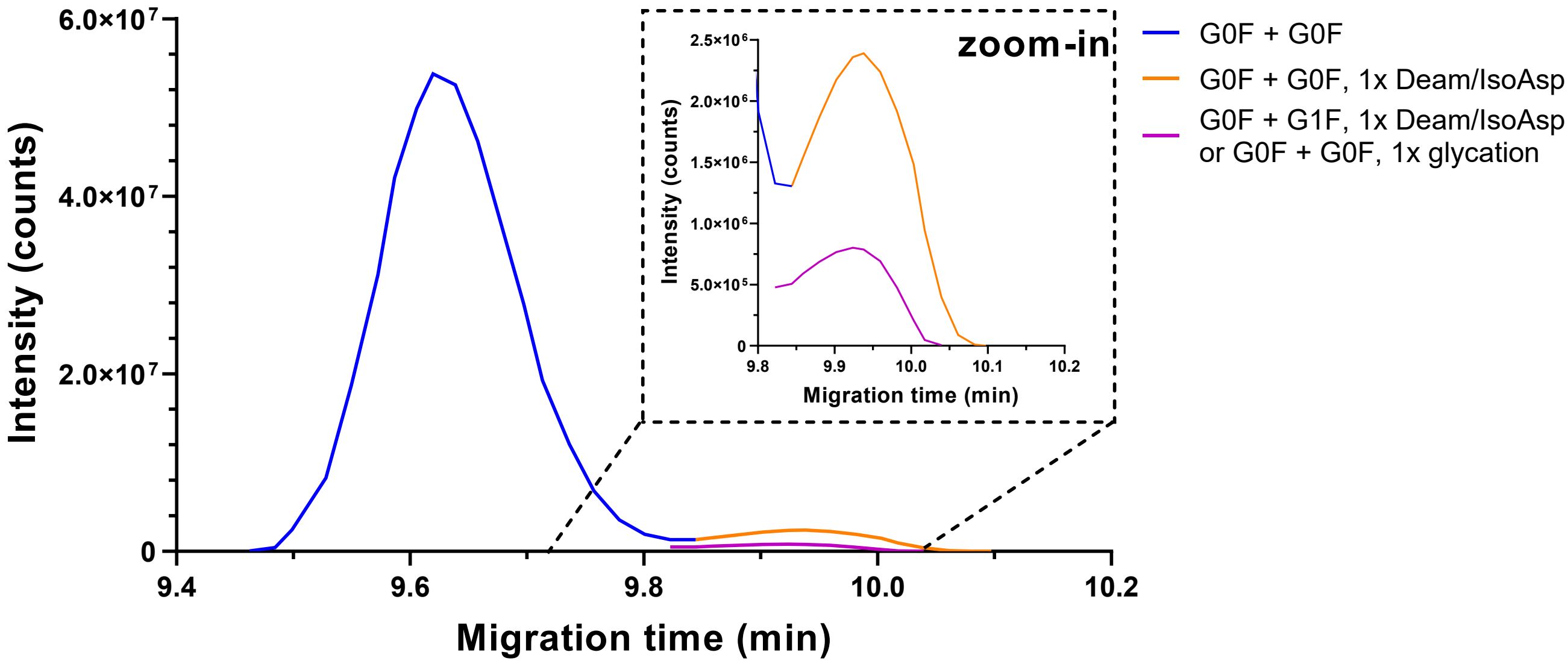

Fig. S6 Extracted ion electropherograms (EIEs) obtained after deconvolution with ProSight Native of the originator sample, obtained with the high-resolution MCE-MS method. The EIEs demonstrate how the main proteoform (G0F + G0F, blue trace) is separated by the software from the deamidated and/or isomerized asparagine G0F + G0F proteoform (orange trace) despite their almost similar mass (+0.98 Da theoretical mass difference). The software also found a proteoform in the acidic region with a mass of +162 Da, which could either be assigned to the deamidated and/or isomerized variant of the G0F + G1F proteoform or a single glycation event (purple trace). The zoom-in highlights the acidic-migrating proteoforms

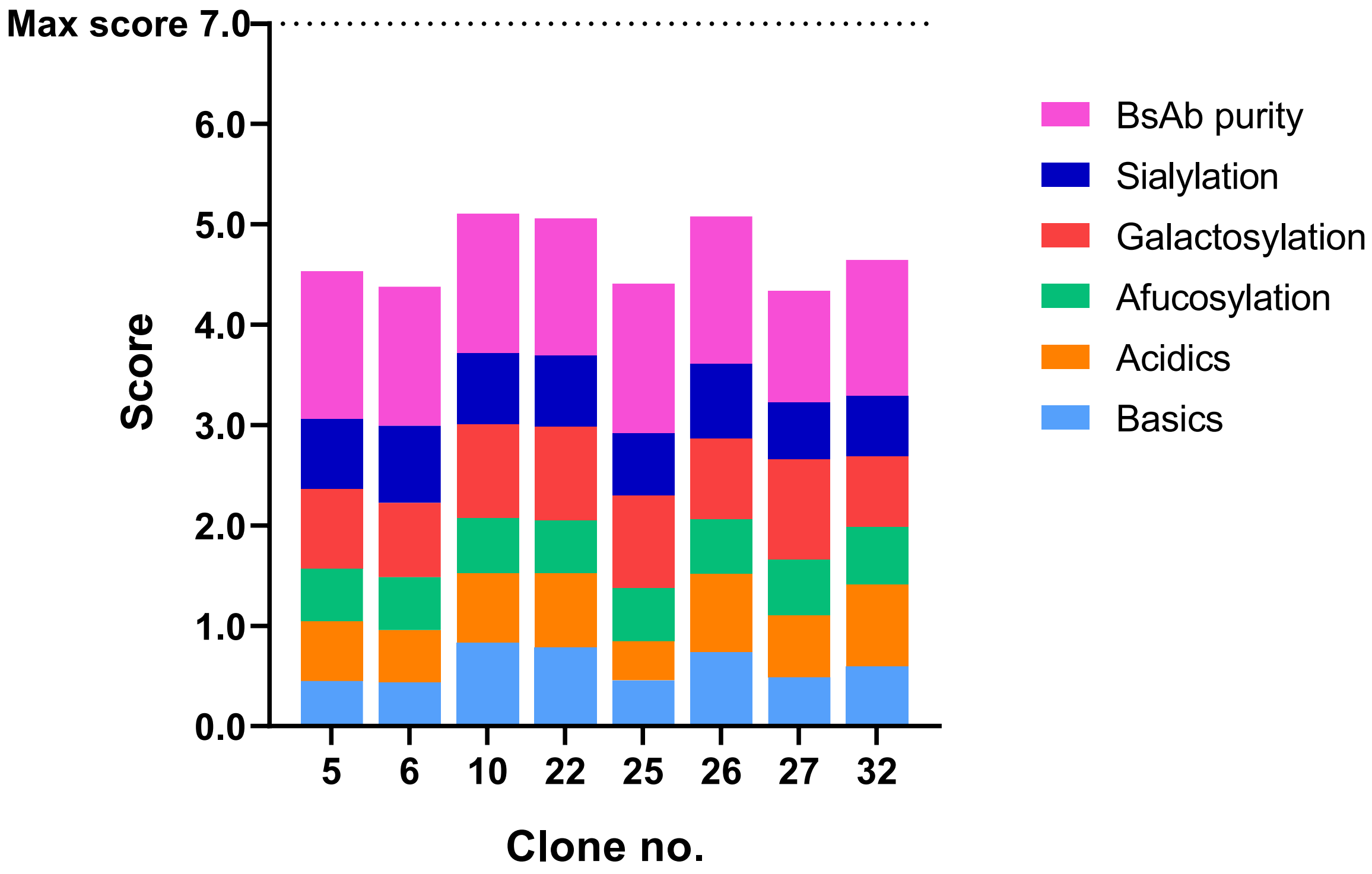

Fig. S7 Bar chart depicting the cell line selection scores for the selected top 8 clone BAbs based on originator similarity in terms of afucosylation, galactosylation, sialylation, BsAb purity, basic variants, and acidic variants as measured using both the high-throughput (for BsAb purity) and high-resolution (for all other outcomes) MCE-MS method. BsAb purity was weighed with a factor of 2, while a weighing factor of 1 was applied for the other outcomes. A maximum score of seven could be attained, which indicated complete similarity to the originator. Scoring procedure is described in ref. [23]

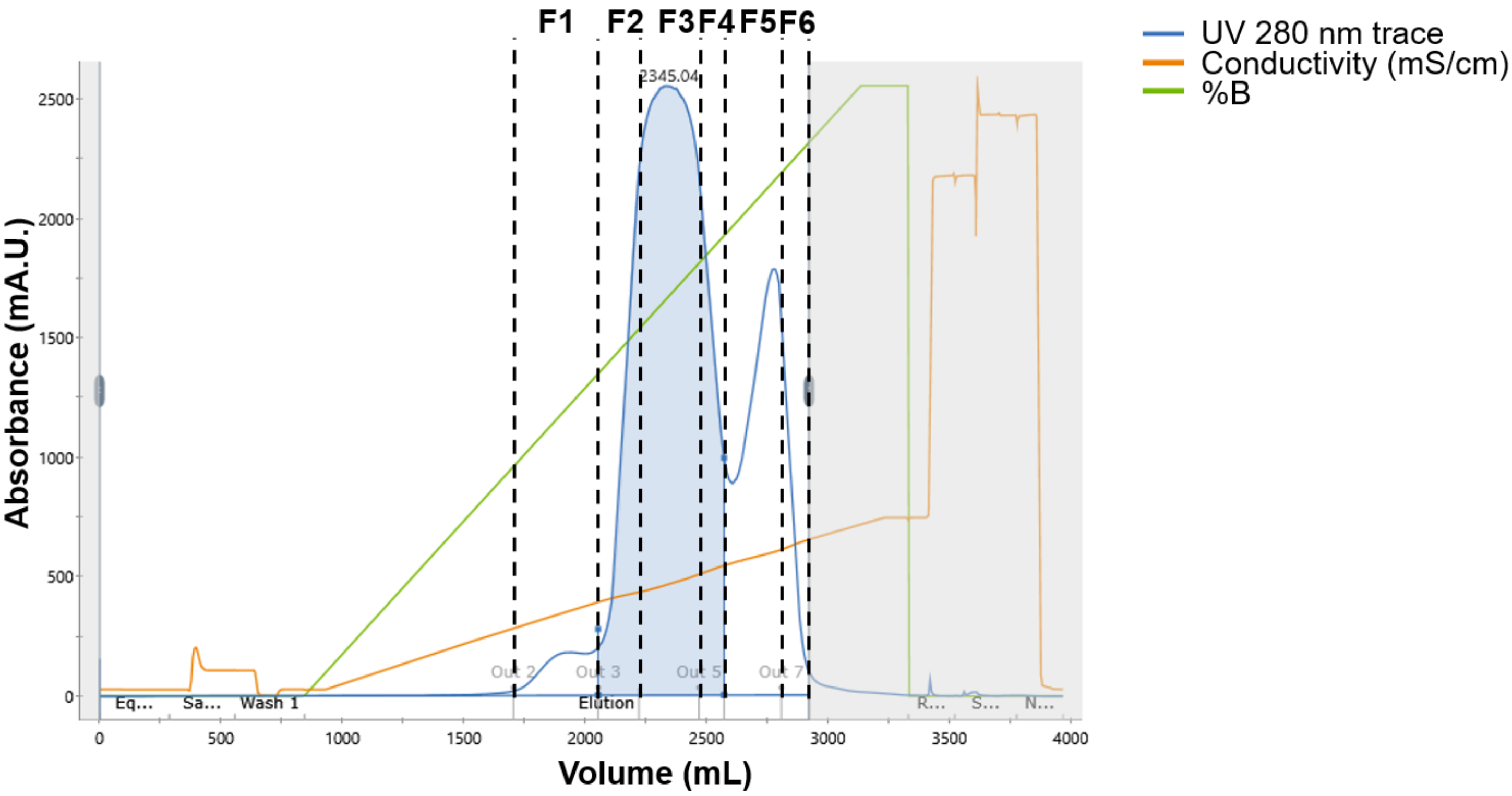

Fig. S8 Preparative cation exchange chromatography (CEX)-UV chromatogram with the blue trace indicating the absorbance at 280 nm, the orange trace indicating the conductivity (mS/cm) and the green trace indicating the %B solvent (increase in salt concentration). The peaks in the chromatogram were fractionated, as specified by the dotted lines and denoted F1 till F6. The highlighted peak between F2-F4 corresponds to the BsAb

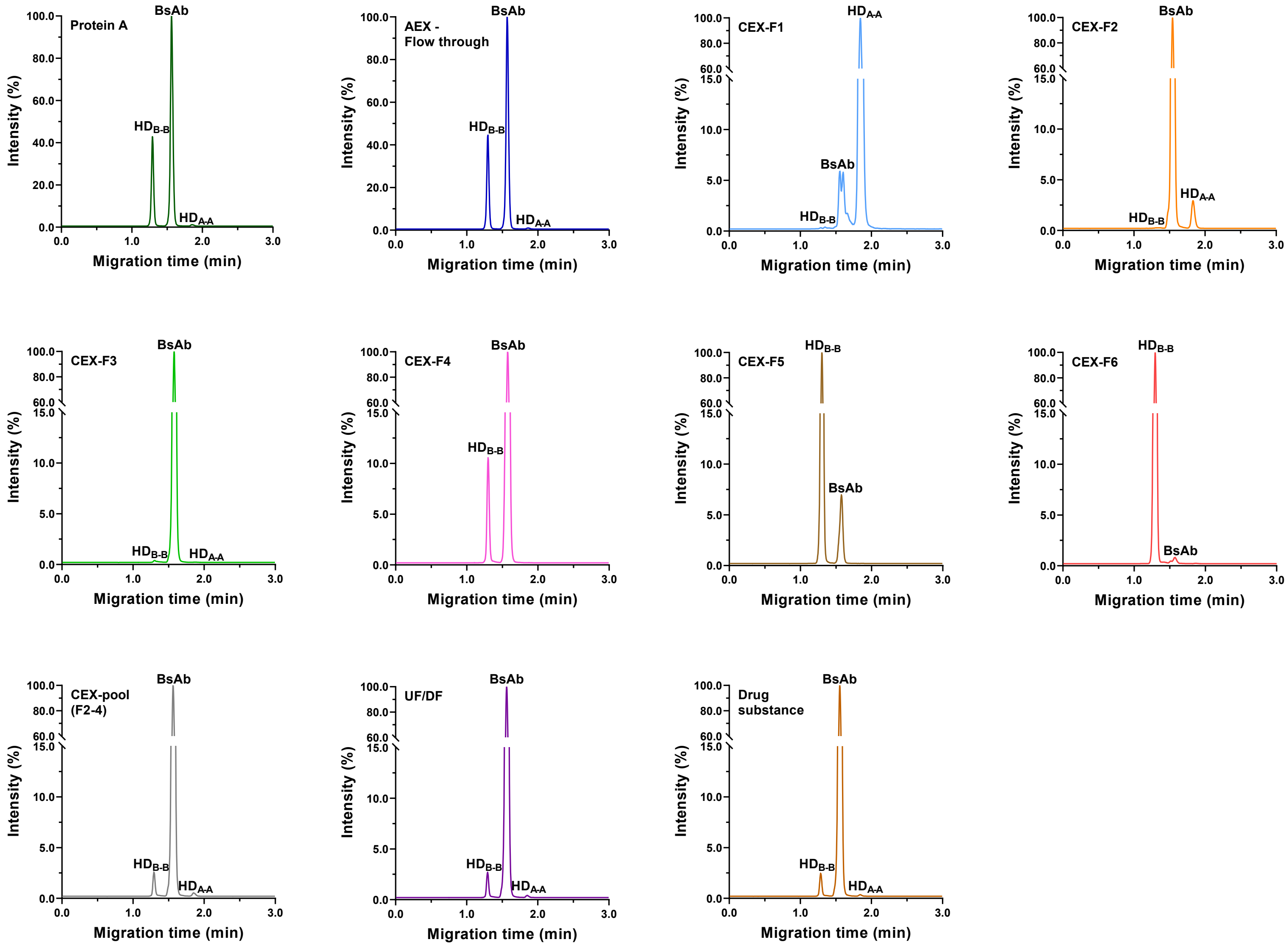

Fig. S9 BPEs depicting all tested DSP process intermediate samples, obtained with the high-throughput MCE-MS method

Table S1 Migration time reproducibility calculated for the BsAb and the homodimers HD_{A-A} and HD_{B-B}, obtained with the high-throughput MCE-MS method. The average (min), standard deviation (min) and relative standard deviation (RSD) are determined for the calibration curve samples and the spiked samples, as described in Section 3.2

Sample set		HDB-B	BsAb	HDA-A
**Calibration curve (n=18)**	**Average (min)**	1.23	1.45	1.76
	**Stand. dev. (min)**	0.04	0.05	0.01
	**RSD (%)**	3.62	3.63	0.57
**Sample A (n=3)**	**Average (min)**	1.20	1.44	1.69
	**Stand. dev. (min)**	0.00	0.00	0.01
	**RSD (%)**	0.22	0.20	0.71
**Sample B (n=3)**	**Average (min)**	1.21	1.45	1.71
	**Stand. dev. (min)**	0.01	0.01	0.00
	**RSD (%)**	0.42	0.52	0.29
**Sample A + B (n=6)**	**Average (min)**	1.20	1.45	1.70
	**Stand. dev. (min)**	0.01	0.01	0.02
	**RSD (%)**	0.61	0.70	0.98

Table S2 Fractional abundances (%) of all identified glycoforms in the selected top 8 clone and originator BsAbs, obtained with the high-resolution MCE-MS method. Alternative glycoforms with isobaric masses are shown in parentheses. 'N' refers to a GlcNAc moiety

Glycoform	Clone 5	Clone 6	Clone 10	Clone 22	Clone 25	Clone 26	clone 27	Clone 32	Originator
**G0F**	0.0%	0.0%	0.5%	0.0%	0.0%	0.3%	0.5%	0.9%	0.4%
**M5 + M5**	0.0%	0.0%	0.0%	0.0%	0.0%	0.0%	0.0%	0.0%	2.2%
**G0F-N + G0F-N**	0.0%	0.0%	0.0%	0.0%	0.0%	0.0%	0.8%	0.0%	0.0%
**G0 + G0F**	0.0%	0.0%	0.0%	0.0%	0.0%	0.0%	0.0%	0.0%	0.5%
**G0F-N + G0F**	0.8%	1.5%	1.3%	1.4%	3.2%	1.6%	3.2%	1.0%	2.5%
**G0F + G0F**	62.4%	64.9%	62.1%	60.5%	53.7%	63.3%	58.9%	69.1%	57.6%
**G0F + G1F**	11.7%	13.6%	16.6%	14.5%	16.8%	14.8%	17.1%	9.0%	12.5%
**G0F + G0F+N**	13.5%	10.8%	7.5%	10.0%	8.0%	10.0%	6.3%	12.2%	7.4%
**G1F + G1F**<br>(G0F + G2F)	0.3%	0.3%	3.7%	3.8%	5.6%	0.5%	3.6%	0.0%	6.3%
**G0F + G1F+N**<br>(G0F+N + G1F)	4.7%	4.0%	3.2%	3.8%	2.8%	3.5%	2.4%	3.4%	0.0%
**G1F + G1F+N**<br>(G0F+N + G2F or G2F+N + G0F)	2.2%	0.7%	1.1%	1.3%	3.3%	1.2%	1.6%	1.2%	0.0%
**G1F + G2F**	1.3%	1.4%	1.3%	0.8%	3.8%	1.7%	3.4%	2.1%	3.9%
**G1F + G2F+N**<br>(G1F+N + G2F)	0.0%	0.0%	0.0%	0.3%	0.0%	0.0%	0.0%	0.0%	1.6%
**G2F + G2F**	1.1%	0.0%	0.5%	1.4%	1.5%	0.4%	1.7%	0.0%	1.2%
**G0F + G1FS1**	2.2%	2.8%	1.7%	1.7%	1.4%	2.0%	0.6%	1.2%	0.6%
**G1F + G1FS1**	0.0%	0.0%	0.6%	0.6%	0.0%	0.7%	0.0%	0.0%	0.8%
**G1F + G2FS1**<br>(G1FS1 + G2F)	0.0%	0.0%	0.0%	0.0%	0.0%	0.0%	0.0%	0.0%	0.8%
**G1FS1 + G2FS1**<br>(G1F + G2FS2)	0.0%	0.0%	0.0%	0.0%	0.0%	0.0%	0.0%	0.0%	0.6%
**G2F + G2FS1**	0.0%	0.0%	0.0%	0.0%	0.0%	0.0%	0.0%	0.0%	0.3%
**G2FS1 + G2FS1**<br>(G2FS2 + G2F)	0.0%	0.0%	0.0%	0.0%	0.0%	0.0%	0.0%	0.0%	0.4%
